# Cancer Neuroscience: Decoding Neural Circuitry in Tumor Evolution for Targeted Therapy

**DOI:** 10.1002/advs.202506813

**Published:** 2025-09-09

**Authors:** Mengyu Yuan, Rongjiao Xi, Yong Kang, Ming‐jie Kuang, Xiaoyuan Ji

**Affiliations:** ^1^ State Key Laboratory of Advanced Medical Materials and Devices Medical College, Tianjin University Tianjin 300072 China; ^2^ Department of Orthopedics Shandong Provincial Hospital Affiliated to Shandong First Medical University Jinan Shandong 250021 China

**Keywords:** cancer neuroscience, cancer therapy, nervous system, neural–tumor interaction, tumor microenvironment

## Abstract

Recent breakthroughs in tumor biology have redefined the tumor microenvironment as a dynamic ecosystem in which the nervous system has emerged as a pivotal regulator of oncogenesis. In addition to their classical developmental roles, neural‒tumor interactions orchestrate a sophisticated network that drives cancer initiation, stemness maintenance, metabolic reprogramming, and therapeutic evasion. This crosstalk operates through multimodal mechanisms, including paracrine signaling, electrophysiological interactions, and structural innervation guided by axon‐derived guidance molecules. Key discoveries reveal that tumors actively recruit and remodel local neurons, hijacking neurodevelopmental pathways to foster invasive growth. Moreover, malignant cells exhibit neuronal‐like plasticity, adopting electrophysiological properties that increase survival under therapeutic stress. These findings position neural mimicry as a hallmark of aggressive cancers. The expanding field of cancer neuroscience seeks to unravel the essential signaling factors that drive the complex communication between cancer and the nervous system, utilizing these findings to enhance precision therapies for cancer management. In this Review, we highlight considerable advancements in cancer neuroscience studies, sparking further discussions on various research possibilities and outlining a direction for future investigations. Additionally, we explored promising therapeutic strategies rooted in neural–tumor interactions that could synergize with conventional standard treatments, offering renewed therapeutic vigor for many refractory malignancies.

## Graphical abstract

This review highlights the pivotal role of the nervous system in cancer progression, emphasizing how neural–tumor interactions—via synaptic signaling, neurotransmitters, and paracrine factors—reshape the tumor microenvironment. It introduces cancer neuroscience as an emerging field and proposes integrating advanced tools such as AI and optogenetics to develop precision therapies targeting neural circuits for improved cancer treatment.

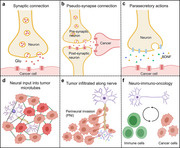

## 1 Introduction

Throughout the entirety of life, the nervous system, which is essential for all physiological functions of the organ system, orchestrates the development of tissues and organs, maintains homeostasis, and regulates plasticity and regeneration. Despite its extensive and pivotal role within the human body, the important role of the nervous system in cancer has long been overlooked. Its branches pervade the entire body, often accompanying the microvasculature and penetrating nearly all tissue microenvironments, saving the epidermis. Similarly, this dense neural innervation reaches the tumor microenvironment, significantly impacting cancer through mechanisms such as paracrine signaling and electrochemical communication. Thus, the nervous system may play a critical role in various cancers, influencing their onset and progression. Conversely, tumors can reshape neural circuits, potentially causing neurological dysfunctions that further exacerbate cancer progression, creating a vicious cycle.^[^ ^1^ ^]^ Recent studies have increasingly highlighted a profound connection between the nervous system and cancer, giving rise to the field of cancer neuroscience. This interdisciplinary arena, bridging neuroscience and cancer biology, holds promise for advancing the treatment of malignancies that severely threaten human health through future collaborative efforts across multiple disciplines.

First described in the 1930s by Hans Scherer, invasive brain cancer cells enveloping the neuronal cell bodies and dendrites—termed “perineuronal satellite status”—mirrored the clustering of major glial cells (astrocytes and oligodendrocytes) around neurons in a healthy nervous system.^[^ ^2^ ^]^ Scherer research has explored primarily the relationships between brain cancer and its surrounding neurons, laying foundational insights for subsequent studies on neuronal involvement in cancer pathogenesis. Since 2015, landmark discoveries have significantly advanced the field of cancer neuroscience. Within the tumor microenvironment (TME), a substantial number of noncancerous host cells—including cancer‐associated immune cells, endothelial cells, fibroblasts, and neurons—are present.^[^ ^3^ ^]^ Previously regarded mere bystanders, these host cells are now understood to play pivotal roles in the pathogenesis of cancer, such as supplying metabolic products to tumors and assisting in their evasion of immune surveillance.^[^ ^4^ ^]^ Despite extensive research on cancer‐associated fibroblasts (CAFs),^[^ ^5^ ^]^ endothelial cells,^[^ ^6^ ^]^ and immune cells,^[^ ^7^ ^]^ neurons, as critical components of the TME, have garnered less attention despite potentially playing more complex roles in tumor progression and metastasis. Cancer neuroscience, an emerging interdisciplinary field, focuses on the local and systemic interactions between cancer and the nervous system. There is a pressing need for further scientific investigations to explore these interactions and their underlying mechanisms.^[^ ^8^ ^]^ In this review, we present the key achievements in the field of cancer neuroscience, outline the current state of tumor neurology and identify unresolved issues requiring further investigation. Of particular note is the provision of pathways and strategies for the clinical translation of tumor neuroscience. We believe that interventions from neuroscience and interdisciplinary collaboration will open new perspectives and prospects for cancer treatment in the future, necessitating further development and translation.

**Table 1:** Clinical trials targeting neural–cancer interactions: mechanisms and translational progress.

Tumor Type	Therapeutic Target	Intervention	Key Mechanism and Biological Impact	Clinical Trial ID	Refs.
Pediatric High‐Grade Gliomas	ADAM10/NLGN3 axis	INCB7839 (ADAM10 inhibitor)	Inhibits cleavage of NLGN3, blocking PI3K‐mTOR signaling and glioma cell proliferation	NCT04295759	[28]
Glioblastoma	AMPA receptor‐mediated synaptic integration	Perampanel	Noncompetitive AMPA receptor antagonist; suppresses tumor‐induced seizures and Ca^2^⁺‐driven growth	NCT03636958, NCT00267592	[20, 22]
Breast Cancer Brain Metastasis	NMDA receptor signaling	Memantine	Blocks glutamate‐dependent activation of NMDARs, inhibits metastatic colonization in the brain	NCT04939597	[25]
Recurrent Glioblastoma	Gap junction networks	Meclofenamate and Temozolomide	Disrupts intercellular Ca^2^⁺ communication via gap junctions, enhances chemotherapy efficacy	Eudra‐CT 2021‐000708‐39	[122]
Pancreatic Ductal Adenocarcinoma	β‐Adrenergic signaling	Propranolol	Inhibits stress‐induced sympathetic activation, reduces NGF secretion and tumor progression	NCT03572283	[123]
Prostate Cancer	Autonomic nerve innervation	Botulinum Toxin A	Induces local denervation, suppresses adrenergic‐driven angiogenesis and metastasis	NCT01520441	[124]
Bone Metastases	NGF/TrkA signaling	Tanezumab (Anti‐NGFmAb)	Alleviates neuropathic pain and inhibits tumor‐associated neurogenesis	NCT02609828	[125]
Gastric Cancer	Cholinergic/Muscarinic receptor	Darifenacin	Inhibits Wnt pathway activation by cholinergic neurons, reduces tumor stemness	Preclinical	[44b]
Melanoma	CGRP‐mediated T‐cell exhaustion	CGRP receptor antagonists	Reverses CD8⁺ T‐cell exhaustion induced by sensory neurons, synergizes with anti‐PD1 therapy	Preclinical	[76a]
Pancreatic Adenocarcinoma	Neurite outgrowth regulators	Anti‐NCAM1 antibodies	Blocks Schwann cell‐mediated cancer migration along nerves, inhibits perineural invasion	Preclinical	[126]

Clinical trial data were obtained from ClinicalTrials.gov. and the EU Clinical Trials Register.

## 2 Development and Regulatory Functions of the Nervous System

Neuronal activity orchestrates the development, equilibrium, and plasticity of organs throughout the body, yet cancer emergence and progression hijack this regulatory mechanism. A deeper understanding of neurodevelopment, neuronal circuitry establishment, and plasticity can provide crucial insights into the involvement of neurons in cancer mechanisms.^[^ ^9^ ^]^ The nervous system modulates development, internal balance, and plasticity by regulating the functions of stem cells and progenitor cells.^[^ ^10^ ^]^ Neuronal circuit assembly encompasses axonal growth and pathfinding, synaptogenesis, the refinement of connections between neurons, and the formation of myelin sheaths^[^ ^11^ ^]^ In addition to neurons, essential elements of the nervous system include astrocytes, oligodendrocyte precursor cells (OPCs), microglia, and Schwann cells, among others, whose interactions and coordination constitute a comprehensive and efficient neural control network.^[^ ^12^ ^]^ Astrocytes, a primary type of neuroglial cell, are crucial regulators of synaptic formation and function during development.^[^ ^13^ ^]^ Although neurogenesis precedes the generation of astrocytes in the cortex, synaptic formation commences only after the generation of astrocytes, coinciding with the branching and protrusion of neurons.^[^ ^14^ ^]^ The interaction between neurons and OPCs is paramount for the normal development and formation of myelin sheaths. Brain‐derived neurotrophic factor (BDNF) serves as a crucial secretion protein that regulates the development and function of neural circuits, including processes dependent on neuronal activity in the mammalian brain, such as neuronal differentiation and growth, synaptogenesis and plasticity, and increased cognitive functions.^[^ ^15^, ^16^ ^]^

Typical neuronal actions include the generation of action potentials in resting cells, the transmission of neural impulses, neurotransmitter release leading to membrane depolarization at the electrochemical synapse, and voltage‐dependent calcium influx, all of which influence a spectrum of cellular functions. Neuronal activity is crucial for neuroeducation, proliferation, migration, and differentiation of neural stem and progenitor cells, synaptogenesis, and oligodendrocyte precursor cell genesis.^[^ ^17^ ^]^ Electrical activity permeates every phase of nervous system formation, starting from the initiation of neurons during embryonic development to the adaptability and malleability of intricate neural networks in the mature brain.^[^ ^18^ ^]^ Typically, neurons transmit electrical activity and communicate with their cellular neighbors in several ways: 1) by establishing gap junctions, 2) by forming bona fide synapses, and 3) by releasing neurotransmitters and paracrine factors. Strikingly, tumors coopt these neural communication modalities to drive malignancy, hijacking synaptic architectures to establish proinvasive niches and exploiting neurotransmitter signaling to fuel metabolic adaptation under therapeutic stress.

Taken together, these neurodevelopmental processes, ranging from synaptogenesis and glial maturation to activity‐dependent signaling, are not only fundamental to the construction and refinement of the nervous system but also represent biological pathways that cancers can pathologically exploit. Tumor cells, particularly those in the central nervous system or with strong neurotropic behavior, have been shown to coopt these developmentally programmed mechanisms to promote proliferation, migration, synaptic integration, and resistance to therapy. Therefore, understanding the architecture and logic of normal Neural Development is essential for identifying how cancers rewire neural regulatory circuits to their advantage.

## 3 The Nervous System Drives Tumor Progression

Psychological conditions such as chronic stress, anxiety, and depression have long been recognized as factors influencing cancer progression through neuroendocrine and immune pathways.^[^ ^19^ ^]^ In addition, neurological disturbances, such as neural injury or neurodegeneration, may affect tumor biology via distinct neural mechanisms. This clear distinction highlights the multifaceted role of the nervous system in cancer development.

Mechanistically, the nervous system modulates tumor progression through synaptic neurotransmission, paracrine signaling, and microenvironmental crosstalk, with diverse mechanisms collectively shaping cancer pathogenesis. Critically, the role of the nervous system extends beyond brain tumors to encompass both solid and hematological malignancies. Emerging evidence underscores the necessity of deciphering neural–tumor interactions, as targeting these pathways may offer novel therapeutic strategies to disrupt cancer progression and metastasis.

### 3.1 Central Nervous System‐Driven Tumor Progression

The central nervous system (CNS) consists of the brain and spinal cord and serves as the primary control center of the body. It processes sensory information, coordinates motor functions, and governs cognitive and emotional activities. The CNS is essential for integrating neural signals and maintaining homeostasis, forming the foundation for overall neurological function and behavior. In the CNS, the interactions between nerves and tumors are close, and the regulatory role of nerves in tumors is more apparent. The nervous system regulates the growth, metastasis, and treatment response of tumor cells through various pathways. There is a complex interplay between nerves and tumors, and gaining a deeper understanding of this relationship will be highly important for tumor research and treatment.

#### 3.1.1 Direct or Indirect Synaptic Connections

**Figure 1:**
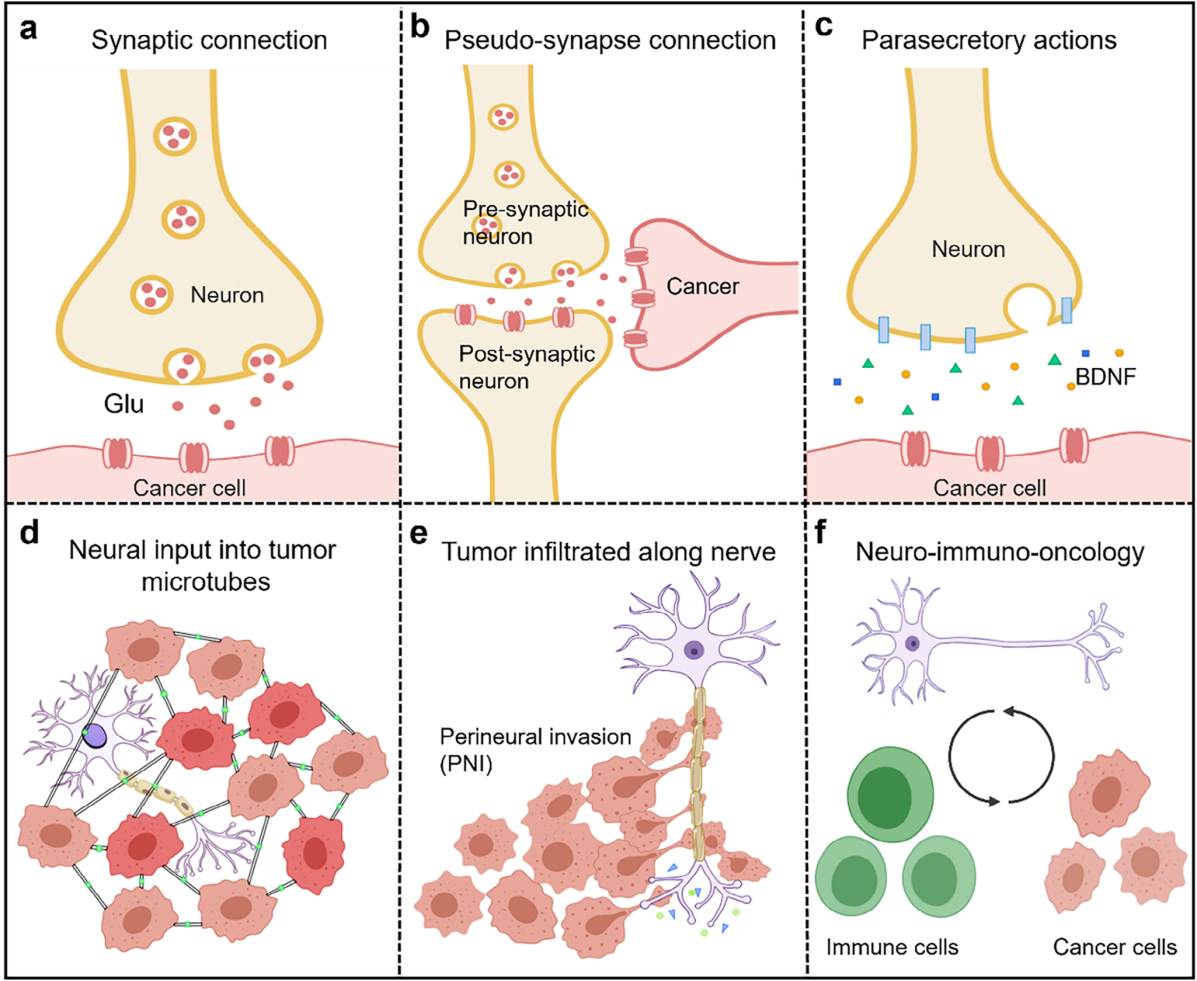
Multiple mechanisms of nerve–tumor interactions. a) Synaptic connections between neurons and tumor cells. b) Pseudosynapses. c) Neurons secrete para‐secretory factors that act on tumors. d) Tumor cells form a network of functions and resistances to which nerves are input. e) Tumor cells infiltrated along the nerve. f) The nervous system acts on tumors through immune cells.

**Figure 2:**
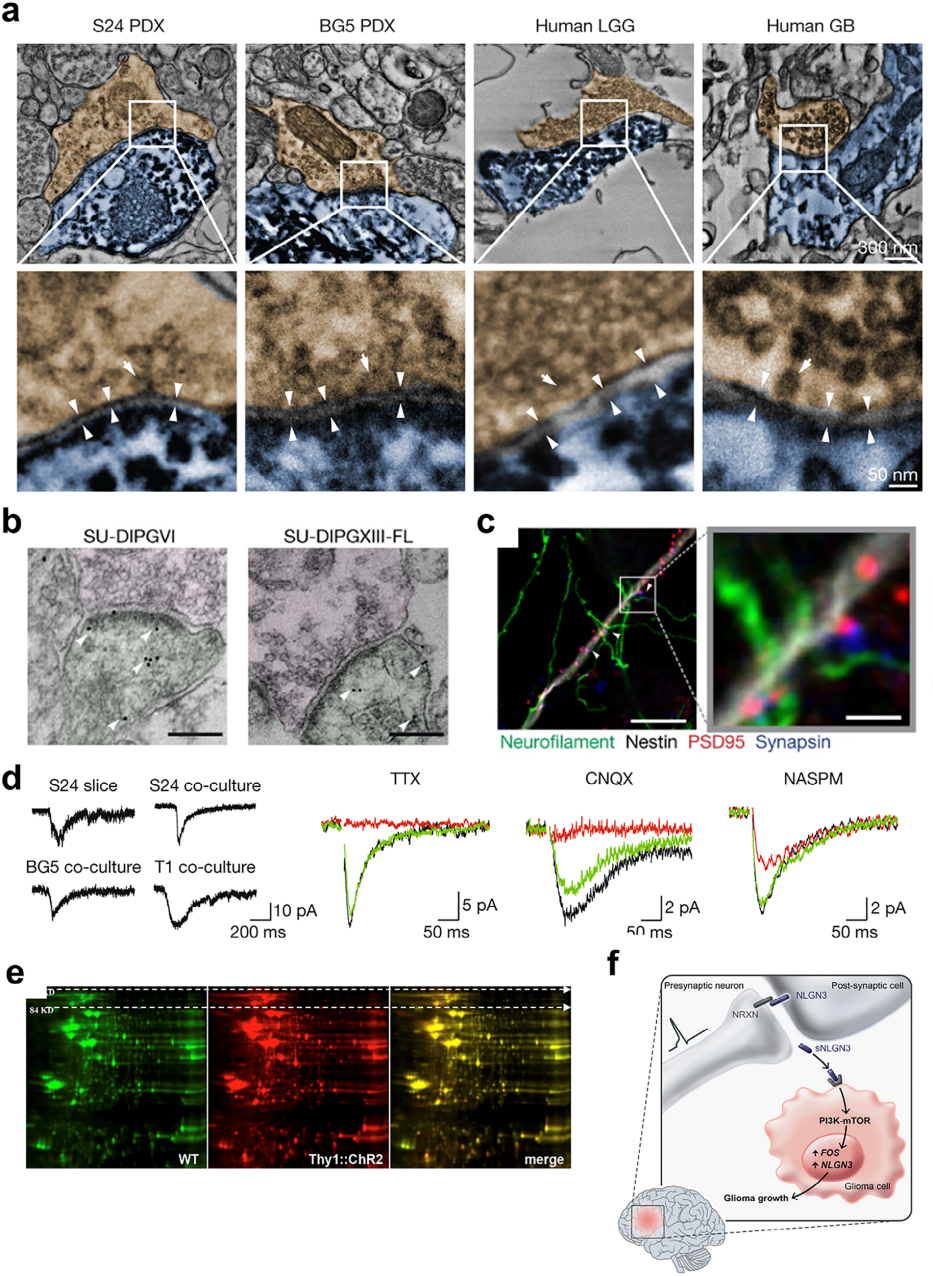
a) Immunoelectron microscopy reveals synaptic connections between presynaptic axons and postsynaptic glioma cells.^[^ ^20^ ^]^ Copyright 2019, Springer Nature. b) Immunoelectron microscopy analysis of synaptic interactions in patient‐derived DIPG (diffuse intrinsic pontine glioma) xenografts. c) Confocal microscopy analysis of synaptic connectivity between neurons and glioma cells.^[^ ^22^ ^]^ Copyright 2019, Springer Nature. d) Pharmacological agents suppress neuronal‐induced excitatory synaptic currents and delay inward potassium currents in GBM cells.^[^ ^20^ ^]^ Copyright 2019, Springer Nature. e) 2D gel electrophoresis demonstrated specific enrichment of NLGN3 in tumor‐associated neural secretions. f) Schematic model showing how synaptic activity induces ADAM10‐mediated cleavage of NLGN3 from postsynaptic neurons, releasing it into the tumor microenvironment. Secreted NLGN3 binds glioma cell receptors, activating PI3K‐mTOR signaling. This pathway stimulates oncogenic FOS expression and further NLGN3 production, ultimately driving tumor cell proliferation.^[^ ^27^ ^]^ Copyright 2015, Cell Press.

In primary CNS gliomas, neurons directly activate tumor cells via synaptic connections, specifically the α‐amino‐3‐hydroxy‐5‐methyl‐4‐isoxazolepropionic acid (AMPA) receptor signaling pathway. In contrast, in CNS metastatic tumors (such as breast cancer brain metastasis), tumor cells may retain the neurodependence of their primary site (such as *N*‐methyl‐d‐aspartate receptor (NMDAR) signaling)), but their integration into the CNS neural network is limited by the blood–brain barrier, which results in a clear difference from the synaptic integration mechanisms observed in gliomas. Direct synaptic connections in the central nervous system have been observed through electron microscopy in patients with intractable gliomas (**Figure** **1**a).^[^ ^20^ ^]^ Presynaptic neurons release the neurotransmitter glutamate, which, mediated by calcium‐permeable AMPA receptors, induces excitatory postsynaptic currents (EPSCs) at synapses, causing depolarization and calcium influx in glioma cells, thereby promoting glioma proliferation and invasion (**Figure** **2**a–c).^[^ ^21^ ^]^ This direct synaptic connection between neurons and tumors drives cancer progression, and genetic knockout or pharmacological inhibition of AMPAR reduces glioma cell proliferation and invasion.^[^ ^22^ ^]^ In addition to EPSCs, potassium ions produced by neuronal activity induce delayed currents in glioma cells, which are amplified through gap junctions in the tumor microenvironment, accelerating glioma progression (Figure 2d). Michelle Monje and her team pioneered the discovery of neuronal input into tumors via synapses, demonstrating the significant role of neurons in glioma progression. OPCs are potential source cells for many types of gliomas,^[^ ^23^ ^]^ and similar to immature neurons, OPCs also receive synaptic inputs.^[^ ^24^ ^]^

Moreover, in breast‐to‐brain metastasis, an indirect form of neuron–tumor synapses has been discovered (Figure 1b), where cancer cells are positioned around synapses adjacent to normal neuron–neuron synapses (“pseudo–triad”) and transmit signals through glutamate receptors (NMDARs) on the cancer cell side, driving the progression of brain metastatic breast cancer cells.^[^ ^25^ ^]^ Overall, these findings suggest that neurons in the central nervous system can form direct or indirect synapses with tumor cells and that tumors can promote their proliferation and progression by hijacking neurotransmitters, which means that tumors can exploit neuron‐derived chemical signals, such as glutamate, by expressing corresponding neurotransmitter receptors. Through this mechanism, tumor cells integrate into neural circuits and coopt excitatory synaptic activity to activate progrowth pathways and enhance malignancy.^[^ ^26^ ^]^

#### 3.1.2 Paracrine Signaling

Additionally, paracrine signaling is another crucial mechanism through which the nervous system regulates tumor growth (Figure 1c). Groundbreaking research by Venkatesh and colleagues revealed that in high‐grade glioma, neuroligin‐3 (NLGN3), a synaptic adhesion molecule, is cleaved by disintegrin and metalloproteinase 10 (ADAM10) and released into the tumor microenvironment. NLGN3 subsequently binds to receptors on the surface of tumor cells, activating the PI3K‐mTOR signaling pathway in glioma cells and driving their proliferation and progression (Figure 2e,f).^[^ ^27^ ^]^ By genetically ablating NLGN3 or inhibiting the hydrolytic enzyme ADAM10 to block NLGN3 signaling, the progression of both high‐grade malignant gliomas is halted.^[^ ^28^ ^]^ The synaptic adhesion molecule NLGN3 plays a crucial role in neuron–glioma interactions. In a neurofibromatosis type 1 (NF1)‐related optic pathway glioma mouse model, excessive shedding of NLGN3 in the optic nerve due to NF1 mutation was observed. In mouse models of NF1‐related optic nerve gliomas in which NLGN3 is deficient or NLGN3 shedding is blocked by drugs, optic nerve gliomas fail to form.^[^ ^29^ ^]^

As previously mentioned, the excitatory neurotransmitter glutamate binds to AMPAR receptors on glioblastomas, promoting tumor proliferation and invasion. Glucose‐regulated protein 78 (GRP78) serves as a neuronally active mitogen in glioma cells, promoting their proliferation.^[^ ^27^ ^]^ BDNF not only acts as a paracrine factor that directly stimulates glioma mitosis but also enhances synaptic connectivity and plasticity between neurons and gliomas. These findings indicate that gliomas hijack neuroplasticity processes, whereby neuronal activity not only promotes tumor progression but also strengthens the connection between neurons and tumors.^[^ ^30^ ^]^ In this process, hijacking neuroplasticity refers to the ability of a tumor to coopt activity‐dependent neuronal remodeling, such as synapse formation, axonal growth, and dendritic reorganization, to facilitate its own integration into neural circuits and benefit from sustained excitatory input. In an olfactory bulb glioma mouse model, insulin‐like growth factor‐1 (IGF‐1) has been recognized as a critical paracrine factor regulated by neuronal activity that promotes the growth of gliomas in the olfactory bulb, further confirming the interaction between neurons and gliomas.^[^ ^31^ ^]^

#### 3.1.3 Neural Input to the Tumor Microtubes

In the CNS, glioma cells can also form elongated membrane protrusions known as tumor microtubes (TMs). These TMs interconnect cancer cells via gap junctions, forming a functional and communicative multicellular network through frequent intracellular Ca^2+^ wave communication. This network enhances tumor resistance to various clinical treatments (Figure 1d).^[^ ^32^ ^]^ Glioma cells hijack neurodevelopmental mechanisms to promote the creation of invasive TMs, which connect individual glioma cells into dense multicellular networks. The accumulation of key proteins for membrane tube growth, such as growth‐associated protein 43 (GAP43) and Tweety homolog 1 (TTYH1), is observed at the ends of TMs, reminiscent of growth cones in neurodevelopmental processes. These structures guide neuronal growth and connectivity, playing critical roles in the development and regeneration of the nervous system. Inhibiting GAP43 with small molecules has been demonstrated to disrupt TM development and function, thereby impeding glioma colonization and progression. Importantly, synaptic neuron–glioma connections facilitate Ca^2+^ communication within the glioma network,^[^ ^33^ ^]^ highlighting the critical role of neuronal input in tumor progression.^[^ ^34^ ^]^ The use of gap junction blockers can effectively inhibit tumor proliferation. While the presence of tumor microtubes has been observed in solid tumors outside the central nervous system, the extent of crosstalk between peripheral nerves and tumor microtubes remains unclear. The presence and function of TMs and synaptic neuron–cancer connections represent important areas for future research.^[^ ^35^ ^]^ Further understanding of their roles in tumor progression and resistance mechanisms will be crucial.^[^ ^36^ ^]^

### 3.2 Peripheral Nerves Drive Tumor Progression

**Figure 3:**
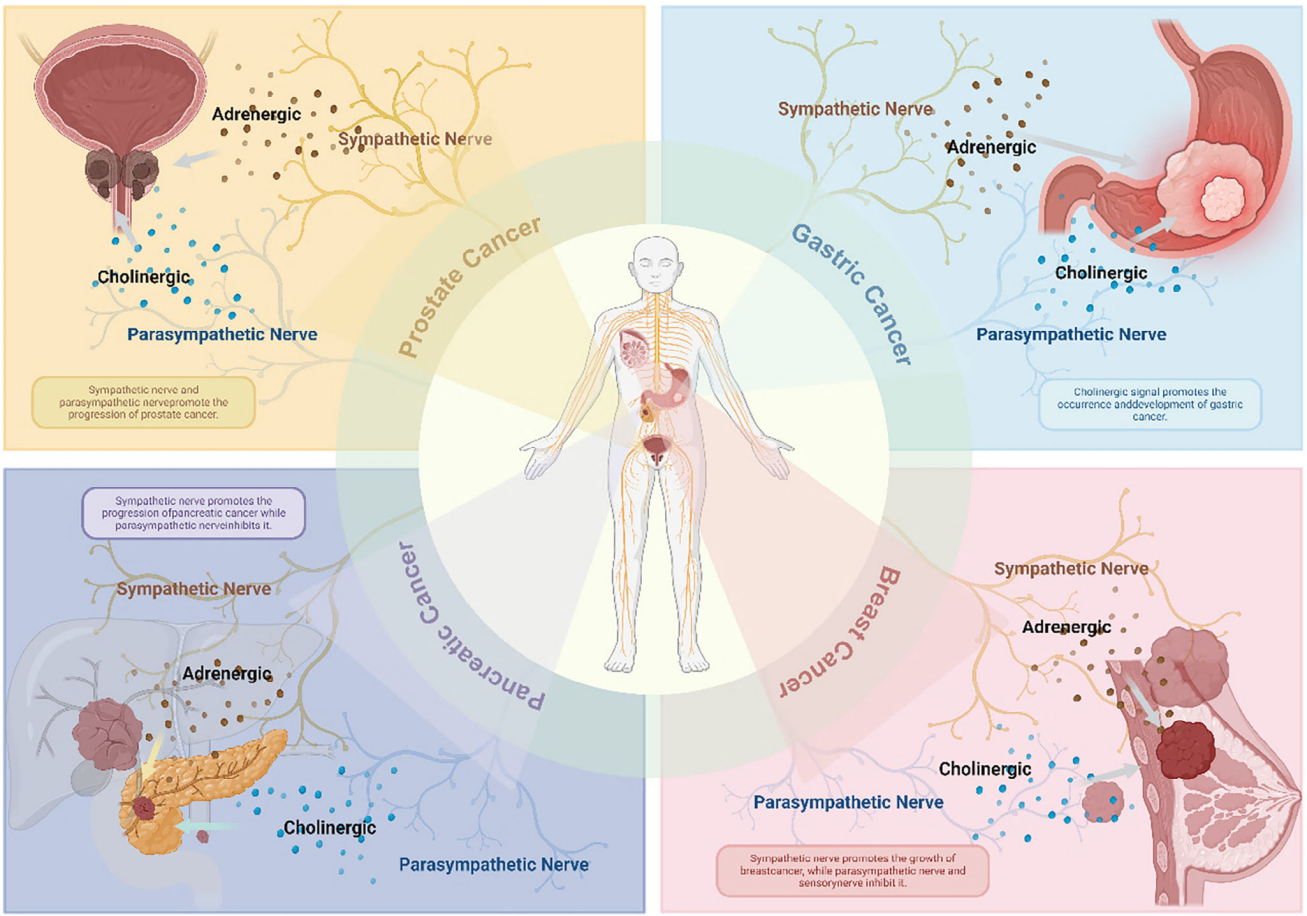
PNS dynamically regulates cancer progression through bidirectional interactions between tumors and neural components. Sympathetic nerves (adrenergic) and parasympathetic nerves (cholinergic) play contrasting roles: Sympathetic nerves drive tumor growth, angiogenesis, and metastasis via β‐adrenergic signaling (e.g., prostate, breast, and pancreatic cancers). Parasympathetic nerves suppress tumorigenesis in pancreatic cancer via cholinergic receptors but promote progression in gastric and colorectal cancers through Wnt signaling.

The peripheral nervous system (PNS) includes all nerves outside the brain and spinal cord and serves as a communication network between the CNS and the rest of the body. It comprises sensory, motor, and autonomic nerves that control tissues and organs. The autonomic nervous system, a key component of the PNS, is divided into the sympathetic (adrenergic) and parasympathetic (cholinergic) branches, which regulate internal organ functions and maintain homeostasis. Early research suggested that the PNS may also drive the onset and progression of systemic cancers. Neural innervation of the PNS autonomic nervous system has been detected in numerous cancers, such as prostate, gastrointestinal, pancreatic, and breast cancers (**Figure** **3**). Unlike primary CNS tumors, the neuroregulation of PNS‐associated tumors (such as prostate cancer and pancreatic cancer) relies more on paracrine signaling from the autonomic nervous system (sympathetic/parasympathetic) rather than synaptic connections. Additionally, although CNS metastatic tumors (e.g., breast cancer brain metastases) are located within the CNS, their neurodependency may reflect characteristics of the primary tumor site and thus require separate analysis.

Early clinical studies revealed another significant manifestation of tumor–neural interactions in the peripheral nervous system: perineural invasion (PNI), where tumor cells invade and spread along nerves (Figure 1e). Signals from neural‐derived neurotrophic and chemotactic factors attract cancer cells, and neural fibers may serve as low‐resistance migration pathways chosen by tumors. Depending on the expression of neural cell adhesion molecule 1 (NCAM1), Schwann cells guide cancer cells to migrate to nerves and promote invasion in a contact‐dependent manner.^[^ ^37^ ^]^ Neuroendocrine macrophages induce the neural invasion of pancreatic cancer cells through the secretion of glial cell line‐derived neurotrophic factor.^[^ ^38^ ^]^ Tumor perineural infiltration is a common feature linked to poor prognosis in various cancers, including head and neck cancer, cervical cancer, biliary tract tumors, gastric cancer, colorectal cancer, pancreatic ductal adenocarcinoma, and prostate cancer.

In this context, we refer to these malignancies as “systemic cancers,” a term denoting tumors that originate outside the CNS yet interact with neural components either locally or distally. When such tumors directly engage with nearby peripheral nerves, particularly through processes such as PNI or autonomic innervation, they are also described as “peripheral tumors” to emphasize their anatomical proximity to and interaction with the peripheral nervous system. This distinction helps clarify that while all peripheral tumors are systemic, not all systemic cancers necessarily exhibit localized neural involvement within the PNS.

#### 3.2.1 Prostatic Cancer

The prostate is abundantly innervated, with both the sympathetic and parasympathetic nerves regulating its development, homeostasis, and function. Early investigations involving prostate surgery to remove nerve innervation have shown significant prostate atrophy.^[^ ^39^ ^]^ These functions of the autonomic nervous system in regulating the prostate suggest a potential parallel role of neurons in prostate cancer. Moreover, histopathological analysis of prostate cancer tissue has revealed PNI.

Groundbreaking research conducted by Frenette and colleagues provides compelling evidence of the sympathetic and parasympathetic neural regulation of prostate cancer.^[^ ^40^ ^]^ Studies have revealed that the presence of autonomic nerve fibers within the prostate plays a pivotal role in modulating the progression and metastasis of prostate cancer in mouse models. Chemical or surgical removal of the sympathetic nerve and gene knockout of β(2)‐ and β(3)‐adrenergic receptors can prevent the early development of tumors. Tumors are also infiltrated by cholinergic fibers of the parasympathetic nerve, which promotes the spread of cancer. Pharmacological blockade or gene destruction of the matrix 1 muscarinic receptor can inhibit cholinergic‐induced tumor invasion and metastasis, thus improving the survival rate of mice. Moreover, analysis of prostate adenocarcinoma samples revealed that the density of sympathetic nerve and parasympathetic nerve fibers in tumors and surrounding normal tissues is related to poor clinical results.^[^ ^40^ ^]^

Research has revealed associations between the development and progression of prostate cancer and other cellular and molecular elements of the nervous system. In orthotopic xenograft and MYC‐driven prostate cancer gene mouse models, β2‐adrenergic signaling stimulates angiogenesis, with reduced oxidative phosphorylation of β2‐adrenergic receptors on endothelial cells in the tumor stroma enhancing tumor angiogenesis.^[^ ^41^ ^]^ Unexpectedly, in a genetically modified mouse model of prostate cancer, neural precursor cells expressing doublecortin migrated from the brain to the prostate cancer microenvironment, generating new adrenergic neurons and subsequently facilitating the onset and progression of prostate cancer, shedding light on the interaction between the central nervous system and prostate cancer.^[^ ^42^ ^]^

#### 3.2.2 Gastrointestinal Cancer

The gastrointestinal system is under extensive autonomic neural control, with regulation from more than 500 million enteric neurons located in the myenteric plexus and submucosal plexus.^[^ ^43^ ^]^ Neurological signals activate the Wnt pathway through multiple mechanisms: cholinergic neurons directly trigger Wnt activation in tumor stem cells via muscarinic acetylcholine receptor 3 (CHRM3), whereas acetylcholine (ACh) secreted by both neurons and chief cells enhances tumorigenesis through a Yes‐associated protein (YAP)‐dependent Wnt‐NGF (nerve growth factor) positive feedback loop. Previous studies revealed that nerve density and Wnt signaling activity are positively correlated with tumor progression and linked to the ACh‐NGF‐YAP‐Wnt axis, which results in self‐sustaining neuro–tumor interactions. Surgical interventions (e.g., vagotomy) and pharmacological inhibitors targeting this axis effectively suppress tumor growth in vivo.^[^ ^44^ ^]^ Notably, elevated NGF and ACh levels alone in gastric stem cell microenvironments are sufficient to initiate tumorigenesis, highlighting the central role of neural involvement. Recent studies have revealed a novel mechanism in gastric cancer: functional synapse‐like connections between peripheral tumors and sensory neurons, resembling synapses in the central nervous system. Tumors recruit sensory nerves via NGF overexpression, forming synapse‐like structures and establishing bidirectional circuits with neurons. These sensory nerves promote gastric cancer growth and metastasis through calcitonin gene‐related peptide (CGRP) and its receptor signaling pathways. CGRP receptor antagonists effectively disrupt tumor–neuron connections, inhibit tumor progression, and prolong survival in murine models.^[^ ^45^ ^]^

Enteric glial cells (EGCs), as critical components of the enteric nervous system, also contribute significantly to the gastrointestinal tumor microenvironment. EGCs not only support and protect enteric neurons but can also be activated by soluble factors released from tumor epithelial cells, which adopt a protumorigenic phenotype. Activated EGCs promote the expansion and tumorigenic potential of cancer stem cells (CSCs) mainly through paracrine secretion of prostaglandin E2, which stimulates EP4 receptors and epidermal growth factor receptor signaling pathways on CSCs. Additionally, tumor‐infiltrating monocytes activate EGCs via IL‐1 receptor signaling, driving EGCs to release interleukin‐6 (IL‐6), which promotes monocyte differentiation into protumorigenic SPP1+ tumor‐associated macrophages.^[^ ^46^ ^]^ This neuroimmune crosstalk between EGCs, immune cells, and tumor cells shapes a favorable microenvironment for tumor progression and immune evasion. Clinically, an increased abundance of EGCs is correlated with poor prognosis in colorectal cancer patients. Moreover, enteric serotonergic neurons produce 5‐hydroxytryptamine (5‐HT), which activates Wnt/β‐catenin signaling in CSCs via specific 5‐HT receptors, further enhancing CSC self‐renewal and colorectal tumorigenesis.^[^ ^47^ ^]^ Together, these findings highlight the complex interplay among enteric neurons, glial cells, and tumor cells within the gastrointestinal cancer niche, revealing novel potential therapeutic targets.

Assessing the effect of neurostimulation on colorectal cancer cells in vitro via a dorsal root ganglion coculture model revealed enhanced neurite outgrowth in human and murine colorectal adenocarcinoma models.^[^ ^48^ ^]^ Moreover, nerve density serves as an independent prognostic factor for colorectal cancer patients and is correlated with overall survival, suggesting that neurogenesis plays a pivotal role in colorectal cancer progression.^[^ ^49^ ^]^ Research has shown that denervation of the sympathetic and parasympathetic nervous systems can inhibit tumor progression in rats treated with the chemical carcinogen DMH (1,2‐dimethylhydrazine), a compound widely used to induce colorectal cancer in preclinical models.^[^ ^50^ ^]^ In the early stages of DMH‐induced carcinogenesis, both sympathetic and parasympathetic denervation have shown efficacy. However, in the late stages, only parasympathetic denervation significantly reduced tumor incidence, tumor volume, and cell proliferation markers. These stage‐dependent effects suggest that parasympathetic innervation plays a distinct role in advanced tumor progression, positioning parasympathetic denervation as a potential targeted therapy for late‐stage colorectal cancer.^[^ ^51^ ^]^

#### 3.2.3 Pancreatic Cancer

**Figure 4:**
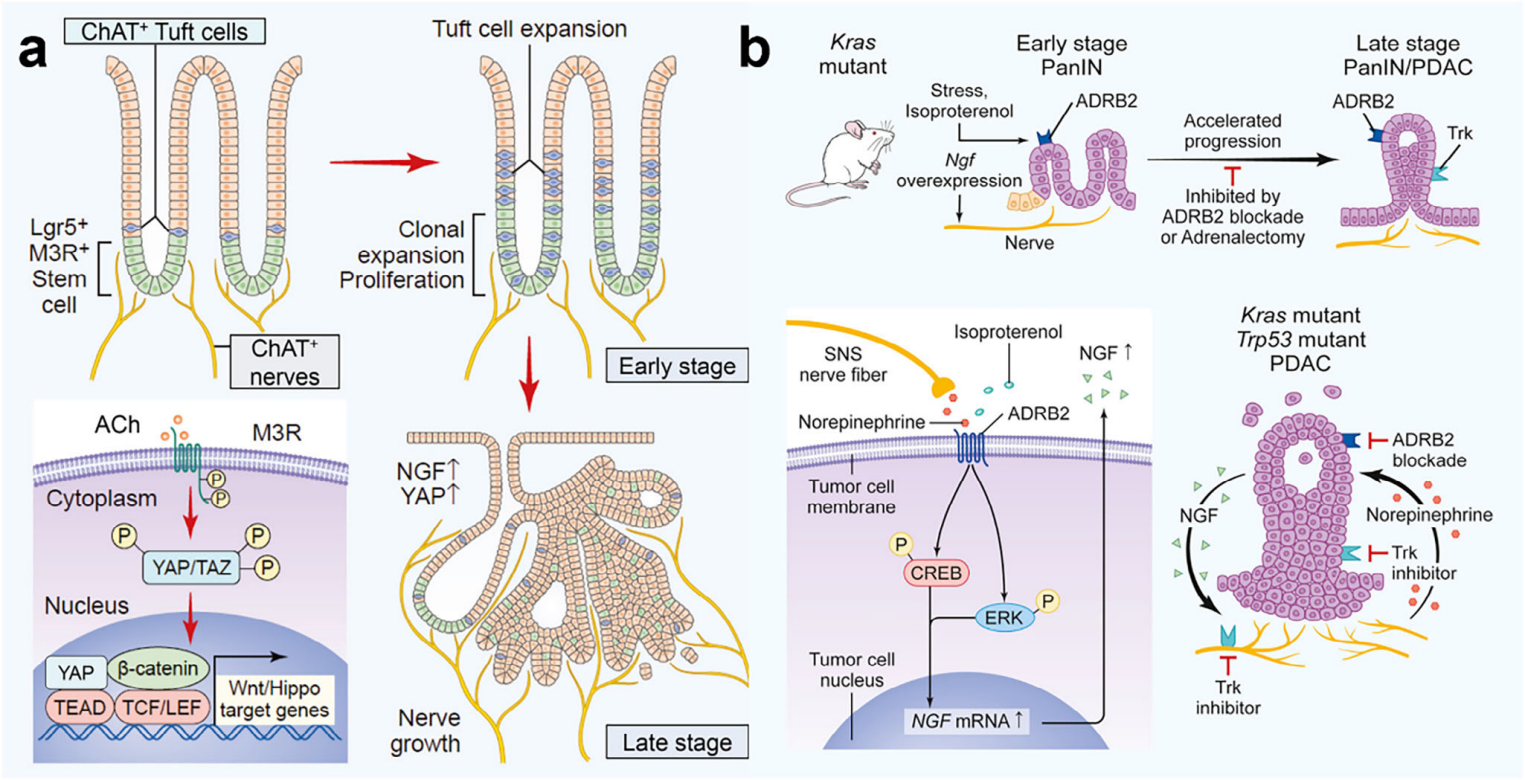
a) Acetylcholine from neurons triggers the release of NGF in gastric epithelial cells, leading to neuronal expansion and tumorigenesis through YAP activation.^[^ ^44b^ ^]^ Copyright 2017, Cell Press. b) Catecholamine promotes the secretion of NGF and then promotes the invasion and malignant proliferation of tumor nerves.^[^ ^58^ ^]^ Copyright 2018, Cell Press.

The pancreas is a nerve‐rich organ surrounded by multiple plexes. Its afferent and efferent nerves regulate not only endocrine and exocrine functions but also visceral reflexes and pain perception. Pancreatic ductal adenocarcinoma (PDAC) frequently presents with pain symptoms closely associated with extensive neural infiltration.^[^ ^52^ ^]^ During early PDAC development, neurotrophic factors such as NGF and its receptor TrkB (tropomyosin receptor kinase B) are markedly upregulated in the pancreas. NGF contributes to PNI, exacerbates cancer pain, and is correlated with tumor progression and prognosis (**Figure** **4**a).^[^ ^53^ ^]^ The Trk inhibitor larotrectinib, which targets tumor‐associated nerves, effectively reduces neural invasion and enhances chemotherapy efficacy.^[^ ^54^ ^]^

Sensory neural innervation plays a crucial role in the initiation and progression of early‐stage PDAC. Nociceptive sensory neurons release CGRP and NGF, which interact with CAFs to suppress interleukin‐15 (IL‐15) expression. This suppresses natural killer (NK) cell infiltration and cytotoxicity, promoting tumor growth and pain.^[^ ^55^ ^]^ Ablation of sensory neurons prevents PNI, neuronal injury, and astrocyte activation, indicating that sensory nerves relay inflammatory signals induced by Kras‐mediated tumorigenesis to the central nervous system.^[^ ^56^ ^]^ Stress‐induced activation of β‐adrenergic signaling promotes tumor growth and dissemination, effects that are reversed by β‐blockers.^[^ ^57^, ^58^ ^]^ Sympathetic catecholamines increase NGF secretion in PDAC cells, stimulate neurite outgrowth, increase nerve density, and accelerate tumor progression.

Conversely, cholinergic signals from the parasympathetic nervous system inhibit PDAC development. Vagal nerve resection in genetic PDAC mouse models leads to increased tumor incidence through the proliferation of malignant CD44^+^ epithelial cells in the tumor stroma. Cholinergic agonists suppress tumor formation and improve survival primarily via antiproliferative Chrm1 signaling.^[^ ^58^ ^]^ Importantly, the role of parasympathetic nerves in cancer is context dependent and varies across cancer types. While cholinergic signaling suppresses tumor progression in PDAC, its effects on gastric and colorectal cancers are contrasting and even promote tumor growth. This highlights the complexity of neural regulation in cancer and cautions. These divergent outcomes likely arise from differences in the tumor microenvironment, neural subpopulations, and downstream signaling pathways specific to each cancer type.

#### 3.2.4 Breast Cancer

The breast harbors a significant amount of adrenergic sympathetic neural innervation, which plays a crucial role in breast cancer. Signaling between the neurotrophic factor BDNF derived from breast cells and the sensory neuron receptor TrkB is essential for establishing sensory neural innervation in the female breast. Testosterone inhibits the BDNF–TrkB signaling axis in sensory neurons, demonstrating a sex‐dimorphic mechanism guiding organ development.^[^ ^59^ ^]^

In the context of cancer, this crosstalk between the nervous system and the breast regulates the progression of breast cancer. Analysis of human breast cancer patient samples revealed that neurotropic invasion is positively correlated with disease progression, metastasis, and clinical stage. Previous studies revealed that coculturing rat neurons with human breast cancer cells resulted in increased secretion of NGF by cancer cells, which stimulated greater neurite outgrowth in adjacent nerves.^[^ ^60^ ^]^ In a preclinical breast cancer model, activation of the sympathetic nervous system drives the progression of breast cancer, which is mediated by norepinephrine synthesized by sympathetic nerves in breast tumors.^[^ ^61^ ^]^ In response to this mechanism, recent research has developed polyethylene glycol lipid nanoparticles loaded with the nonopioid analgesic drug bupivacaine, which can specifically target neurons within tumors. The nanoparticles inhibited neuronal activity by blocking local voltage‐gated sodium channels, significantly reducing nerve‐tumor interactions, and reducing the tumor volume and lung metastasis rate in a triple‐negative breast cancer mouse model.^[^ ^62^ ^]^ In situ human breast cancer xenograft models have shown that specific stimulation of local sympathetic nerve fibers promotes the growth and distant metastasis of primary breast tumors through the release of NE. In contrast, stimulating parasympathetic nerve fibers in the breast cancer microenvironment reduces the growth and distant metastasis of primary tumors. Additionally, there is a synergistic effect between sensory neurons and parasympathetic nerves. The inactivation of sensory neurons induced by capsaicin can enhance the metastasis of breast cancer in mice and make the breast cancer model develop a more aggressive phenotype.^[^ ^63^ ^]^ Recent advances in uncovering molecular plasticity in breast cancer neuroadaptation: Neuronal coculture rapidly induces tumor cell expression of neurotransmitter receptors and synaptic mediators. This neuronal mimicry stratifies brain metastases into GABAergic‐responsive (GRBMs) and dopaminergic subtypes. GRBMs progressively shift from autocrine to paracrine gamma‐aminobutyric acid (GABA) signaling during CNS acclimation, as corroborated by in vivo studies showing that tumor reliance on neuron‐derived GABA is a critical early metastatic strategy. Furthermore, direct neuron–tumor contact epigenetically reactivates Reelin expression via chromatin remodeling, enabling perineuronal niche colonization.^[^ ^64^ ^]^ Taken together, these findings indicate that sympathetic neurons promote breast cancer growth, whereas parasympathetic and sensory neurons inhibit breast cancer growth, highlighting the different regulatory roles that different types of nerves may play in specific tumors. These findings suggest that the effects of nerves on tumors are not singular and that the coordination of positive and negative effects results in complex regulatory effects on nerves.

These breakthrough studies illustrate the key role of the PNS in regulating cancer stem cell niches and driving tumor progression in the feedback loop of nerve–cancer interactions, such as the central nervous system in the context of brain tumors discussed earlier.

### 3.3 Neural Drivers in Nonsolid Tumors

Emerging evidence underscores the critical role of neural components in shaping the pathogenesis and therapeutic resistance of nonsolid tumors, particularly hematologic malignancies. In acute lymphoblastic leukemia (ALL), the CNS microenvironment acts as a sanctuary niche, where direct physical interactions between leukemia cells and CNS‐resident cells (e.g., choroid plexus cells) induce transcriptional reprogramming, including the upregulation of PBX1. This CNS‐driven adaptation enhances chemoresistance (e.g., reduced cytarabine sensitivity) and self‐renewal capacity, contributing to isolated CNS relapse in 30–40% of pediatric ALL patients. Similarly, in acute myeloid leukemia (AML), sympathetic nervous system (SNS) neuropathy disrupts bone marrow niche homeostasis by depleting NG2(+) perivascular cells, which are essential for hematopoietic stem cell (HSC) maintenance, while promoting osteoblast‐primed mesenchymal stromal cell expansion via β2‐adrenergic receptor signaling.^[^ ^65^ ^]^ This adrenergic‐driven remodeling creates a leukemia microenvironment that supports disease progression.

Notably, neural interactions also influence therapeutic outcomes. During CD19 CAR‐T‐cell therapy for B‐cell malignancies, neurotoxicity arises from cytokine storm‐induced endothelial activation (elevated IL‐6, IFN‐γ, and TNF‐α) and blood–brain barrier (BBB) disruption, allowing systemic inflammatory mediators (e.g., IL‐8 and MCP‐1) and CAR‐T cells to infiltrate the CNS. This parallels leukemia's exploitation of CNS niches, as both processes involve BBB permeability and neuroinflammation.^[^ ^66^ ^]^ Furthermore, developmental studies reveal an evolutionary link: embryonic HSC emergence in the aorta–gonad–mesonephros region requires Gata3‐dependent catecholamine production, highlighting the SNS as an ancient regulator of hematopoiesis. Dysregulation of these conserved pathways, whether during development (e.g., Gata3 loss impairs HSC generation) or in adulthood (e.g., SNS‐driven AML niche remodeling), may predispose patients to malignancy or therapy resistance.^[^ ^67^ ^]^

Collectively, these findings position neural systems (CNS and SNS) as key architects of nonsolid tumor ecosystems, driving niche‐specific adaptation, immune evasion, and treatment failure. Targeting neural–tumor interfaces, such as by inhibiting PBX1 in CNS‐infiltrating ALL, blocking β2‐adrenergic signaling in AML, or modulating cytokine‐driven neurotoxicity in CAR‐T‐cell therapy, represents a paradigm shift in combating hematologic malignancies by addressing their neural‐driven vulnerabilities.

### 3.4 Neuro‐Immuno–Oncology

**Figure 5:**
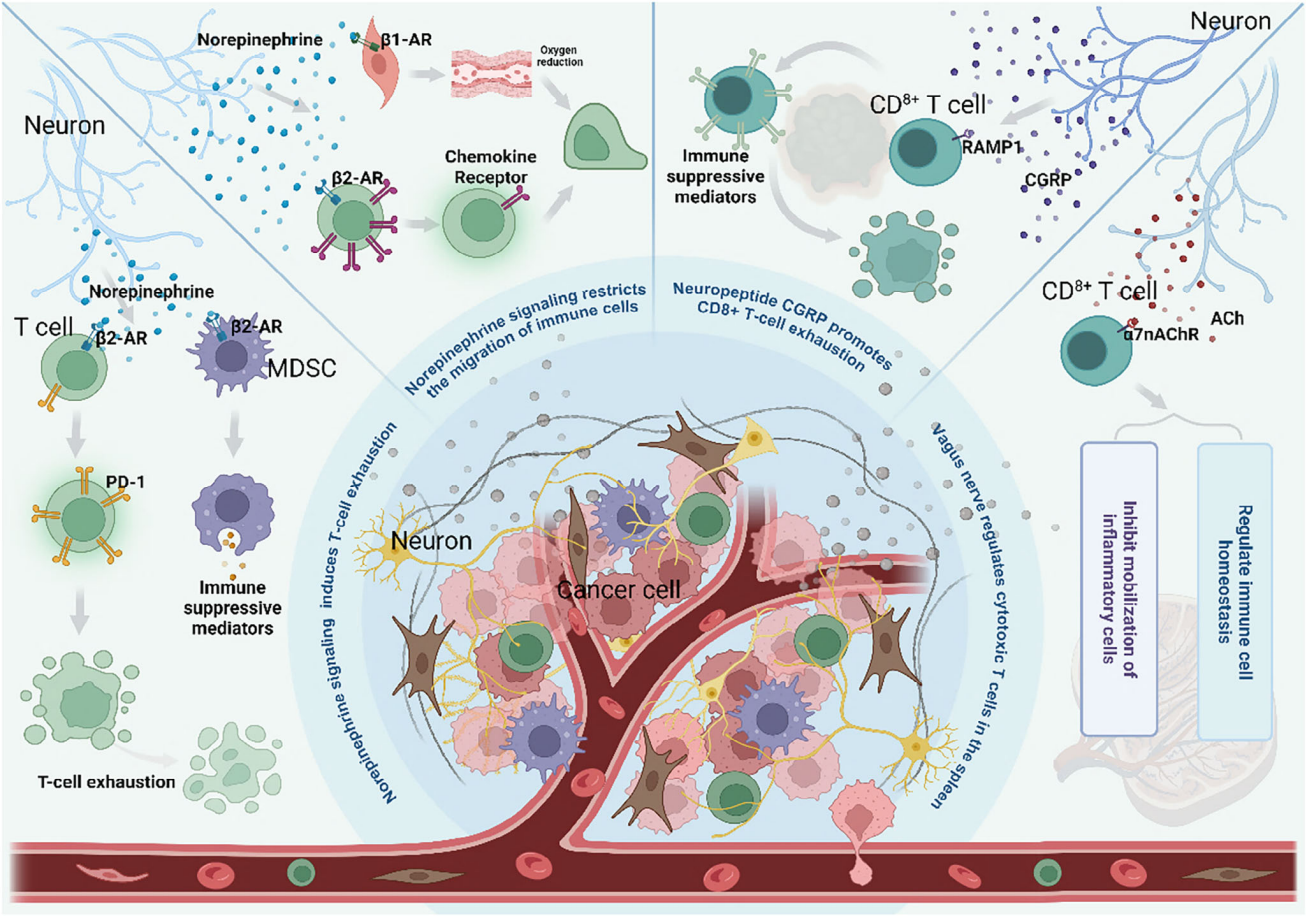
Neuroimmune crosstalk in tumor progression: neural regulation of immunity via neurotransmitter and reflex pathways. The nervous system regulates antitumor immunity through neuroendocrine pathways (e.g., the HPA axis and sympathetic β‐adrenergic signaling), neural reflexes (vagus‐mediated anti‐inflammatory arcs), and direct neurotransmitter interactions (norepinephrine induces T‐cell exhaustion via PD‐1, acetylcholine suppresses inflammation but promotes colorectal cancer, and sensory neuron‐derived CGRP accelerates melanoma).

The nervous system profoundly influences tumor progression by modulating the tumor immune microenvironment, antitumor immunity, and immunotherapy efficacy. This regulatory interplay operates through three principal mechanisms: neuroendocrine pathways, direct neural–immune communication, and neurotransmitter signaling (Figures 1f and **5**).

The hypothalamic–pituitary–adrenal (HPA) axis and the sympathetic nervous system serve as critical bridges between neural and immune activity. Corticotropin‐releasing hormone neurons activate the HPA axis to control monocyte/lymphocyte trafficking between peripheral tissues and the bone marrow.^[^ ^68^ ^]^ Neural reflex circuits, such as the vagus nerve‐mediated anti‐inflammatory pathway, play pivotal roles in modulating tumor immunity. The afferent fibers of the vagus nerve transmit signals related to the peripheral immune status to the central nervous system, whereas its efferent fibers release acetylcholine to inhibit excessive inflammation by modulating cytokine production. This reflex arc maintains immune homeostasis but may also suppress effective antitumor immune responses, thereby facilitating tumor progression (Figure 5). These neuroimmune reflexes constitute a vital regulatory axis within the tumor microenvironment, complementing neuroendocrine and direct neurotransmitter‐mediated mechanisms. Sympathetic β2‐adrenergic signaling governs the lymphocyte circadian rhythm in lymph nodes,^[^ ^69^ ^]^ whereas bone marrow β3‐adrenergic receptors regulate hematopoietic stem cell circulation and inflammatory responses.^[^ ^70^ ^]^ These findings reveal that the neuroendocrine system maintains immune homeostasis through multilevel coordination. The vagus nerve exemplifies bidirectional neuroimmune communication. Its afferent fibers relay the peripheral immune status to the CNS, whereas efferent fibers suppress inflammation via acetylcholine release. This vagal anti‐inflammatory pathway effectively counteracts LPS‐induced cytokine storms in experimental endotoxemia.^[^ ^71^ ^]^ Notably, the adrenergic signal of the sympathetic nerve can also limit the ability of immune cells to migrate between tissues by regulating vascular function, which further expands the regulatory dimension of neural circuits in the immune response.^[^ ^72^ ^]^ The direct regulation of immune cell function by key neurotransmitters is significantly environmentally dependent. For example, norepinephrine enhances the activity of myeloid‐derived suppressor cells through the β2‐adrenergic receptor on the surface of immune cells,^[^ ^73^ ^]^ upregulates PD‐1 expression,^[^ ^19a^ ^]^ and induces T‐cell exhaustion.^[^ ^74^ ^]^ Emerging evidence reveals that β1‐adrenergic signaling drives chronic antigen‐induced CD8+ T‐cell dysfunction. ADRB1 ablation or β‐blockers synergize with checkpoint inhibitors to restore antitumor responses and induce tissue‐resident memory T cells in immunotherapy‐resistant models.^[^ ^75^ ^]^

Similar bidirectional regulatory phenomena also exist in the CGRP signaling pathway. Under physiological conditions, such as during skin injury repair, CGRP‐positive sensory neurons significantly enhance tissue regeneration by recruiting neutrophils and promoting M2 macrophage polarization. However, in the melanoma microenvironment, tumor cells hijack this pathway to inhibit antigen presentation by dendritic cells and upregulate PD‐1 expression on T cells, thereby achieving immune escape.^[^ ^76^ ^]^ Recent studies have further revealed that the widely distributed CGRP‐positive sensory neurons (derived from the T8–T13 dorsal root ganglia) in the spleen promote the formation of germinal centers by activating B cells, suggesting the significant role of this signal in physiological humoral immunity.^[^ ^77^ ^]^ This type of microenvironment‐specific functional transformation provides a theoretical basis for developing therapeutic strategies that selectively block CGRP signals related to tumors.

Therapeutic strategies targeting neuroimmune interactions have demonstrated clinical value. In patients with metastatic melanoma, the combination of nonselective beta‐blockers and immunotherapy significantly improved overall survival and reduced the risk of recurrence.^[^ ^78^ ^]^ Basic research further confirms that the adrenergic signaling pathway directly affects the therapeutic efficacy of immune checkpoint inhibitors by regulating the retention and release of T cells in the lymph nodes.^[^ ^79^ ^]^ These findings collectively point to an emerging therapeutic paradigm: reprogramming neural signals to reshape the immune microenvironment may overcome the current bottleneck of tumor immunotherapy.

## 4 Interference of Tumors in the Nervous System

The interaction between nerves and tumors is not unidirectional. Signals emitted from neurons to cancer cells promote cancer progression, and in turn, tumors can alter or reshape neural circuits, further strengthening carcinogenic signals from neurons.

### 4.1 Tumor‐Induced Nervous System Excitability

**Figure 6:**
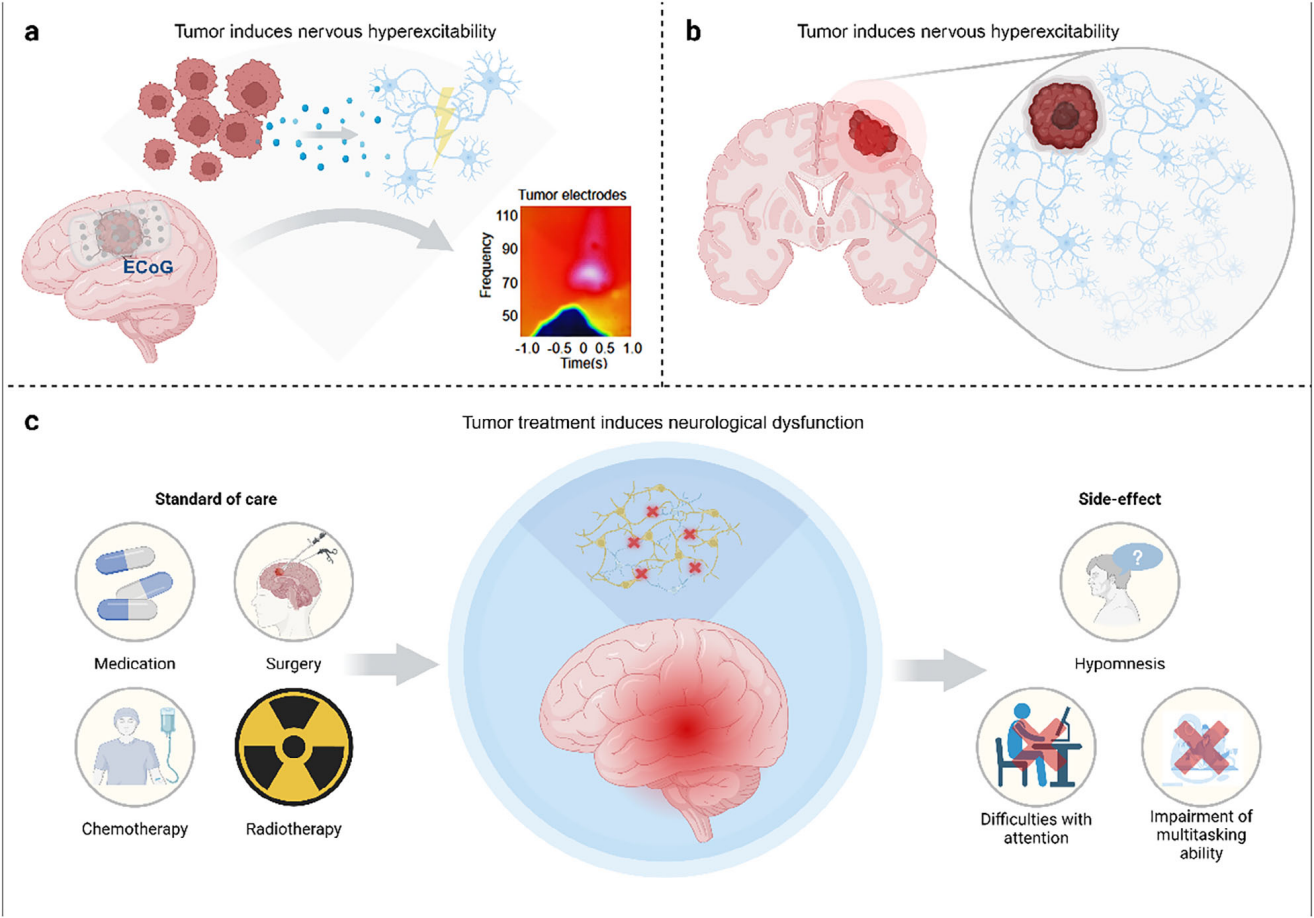
Interference of tumors in the nervous system. a) Tumors induce nervous system hyperexcitability, which can be detected via intracranial ECoG. b) Tumors induce nerve growth. c) Neurological dysfunction caused by tumor treatment, especially chemotherapy and radiotherapy.

**Figure 7:**
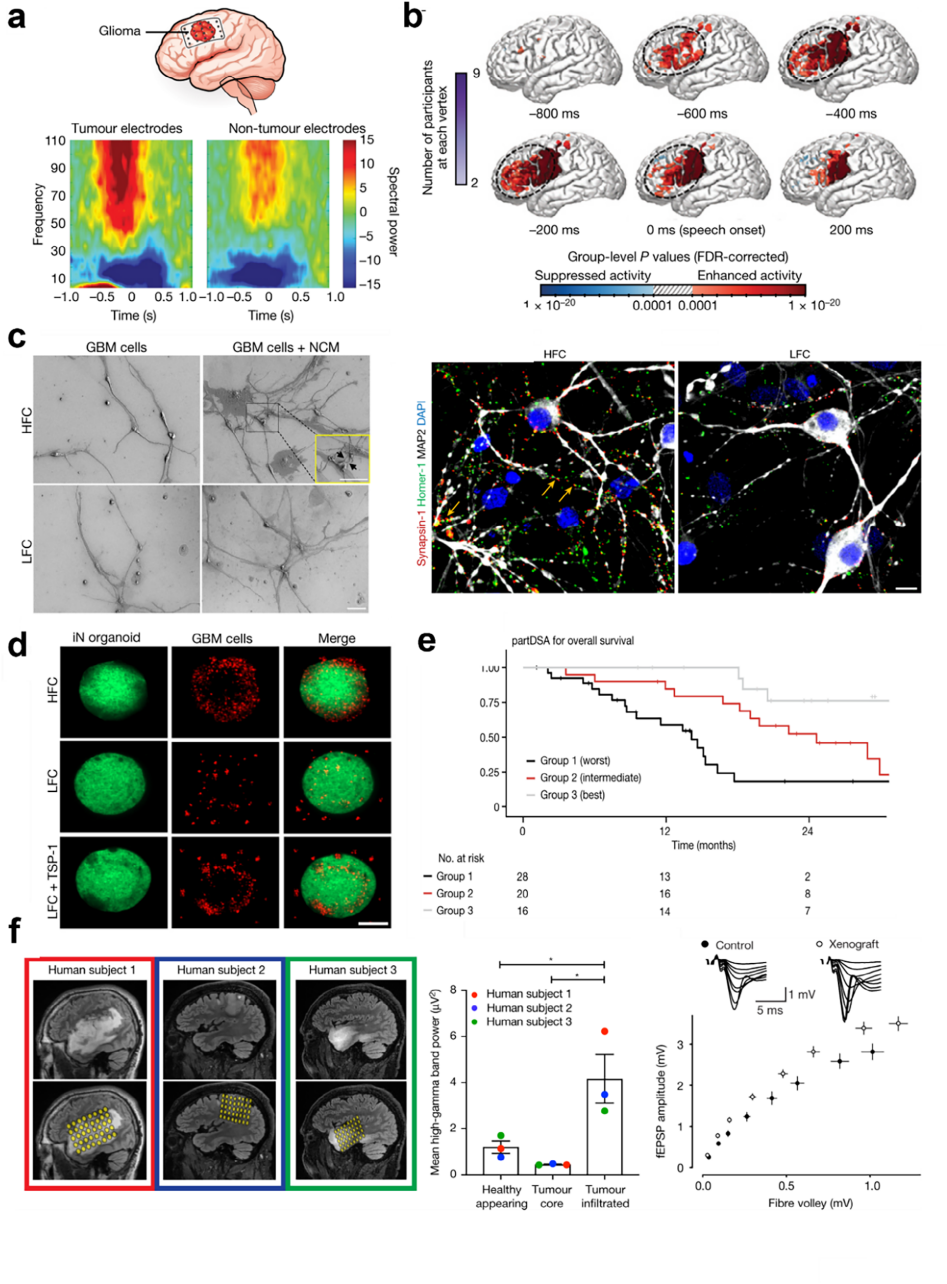
a) In patients with dominant hemisphere glioblastomas, high‐gamma power (HGp, >50 Hz) during audio‐visual speech initiation was recorded via subdural ECoG over the posterior lateral frontal cortex. Spectral analysis revealed distinct frequency separation between tumor and nontumor electrodes, with elevated HGp in tumor‐infiltrated regions. b) Time series of HGp in the tumor‐infiltrated cortex (−600 ms to speech onset, 0 ms), demonstrating dynamic changes in high‐frequency activity preceding speech initiation. This finding suggests that aberrant neural circuit mechanisms—either compensatory or disruptive—may influence language function. c) Glioma reshapes neuronal synaptic networks: differences between synapsin dynamics and TSP‐1‐mediated organoid integration. Intratumoral connectivity is associated with survival and TSP‐1 in patients with high‐grade glioma. d) Fluorescence images showing glioma cells (red) invading neuron clusters (organoids). Adding TSP‐1 (a protein) to LFC glioma cells significantly reduced their invasion ability compared with that of untreated LFC cells. e) Glioblastoma survival risk model. Patients were stratified into high‐ (advanced age/incomplete resection), low‐ (young age/complete resection/no tumor connectivity), and intermediate‐risk groups (complete resection with age/connectivity abnormalities). Survival is jointly determined by age, surgical completeness, and tumor connectivity, highlighting the need to integrate connectivity into clinical risk assessment.^[^ ^82^ ^]^ Copyright 2023, Springer Nature. f) Sagittal MRI localization and intraoperative cortical electrode mapping in IDH‐wildtype glioma patients. ECoG revealed elevated high‐gamma activity in tumor core/infiltrated regions compared with healthy brain regions (*P* < 0.05), suggesting glioma‐driven neuronal hyperexcitability. Electrophysiology in a murine glioma model confirmed enhanced synaptic transmission efficiency (*P* < 0.0001), indicating tumor‐induced synaptic hyperexcitability.^[^ ^22^ ^]^ Copyright 2019, Springer Nature.

In the central nervous system, neuroglial tumors are often accompanied by seizures, which may be related to their direct elevation of neuronal excitability (**Figure** **6**a).^[^ ^80^ ^]^ Neuroglial tumors release glutamate into surrounding tissues, increasing neuronal activity in the microenvironment and leading to neuronal hyperexcitability and triggering seizures. Research has shown that neuroglial tumors increase excitability in the central nervous system through various pathways. For example, neuroglial tumor cells secrete glutamate via the glutamate–cystine exchange system Xc outside synapses.^[^ ^81^ ^]^ Additionally, neuroglial tumor cells secrete factors such as chondroitin sulfate‐3 and thrombospondin‐1 (TSP‐1) (**Figure** **7**).^[^ ^82^ ^]^ Moreover, the number of inhibitory neurons is reduced in the tumor microenvironment, altering neuronal responses to GABA,^[^ ^83^ ^]^ and neuroglial tumors decrease the ability to inhibit GABA currents in the surrounding microenvironment. PIK3CA mutations lead to the secretion of glypican3 by neuroglial tumors, driving neuronal hyperexcitability and synaptic remodeling. The progression of neuroglial tumors and neuronal hyperexcitability form a positive feedback loop, further accelerating tumor progression and neuroconversion.^[^ ^84^ ^]^ In contrast, peripheral system tumors also demonstrate the ability to actively regulate the central nervous system: breast cancer, lung cancer, etc., can activate the paraventricular nucleus of the hypothalamus by secreting leukemia inhibitory factor and galectin‐3, driving excessive activation of the sympathetic nerve axis and then inhibiting antitumor immunity through norepinephrine.^[^ ^85^ ^]^ This remote neural manipulation forms a complementary loop with the malignant progression of peripheral tumors; that is, central tumors trigger epilepsy through local microenvironment remodeling, whereas peripheral tumors promote immune escape through system‐level neural activation. Both confirm the theoretical framework of “tumor–neural system mutualism.”

### 4.2 Tumor‐Induced Neurogenesis

We are aware that many cancers originate from glandular and ductal epithelial tissues. During the normal development of these tissues, cells secrete substances such as neurotrophic factors, which promote the recruitment of nerves, axonal pathfinding, and neurite outgrowth.^[^ ^86^ ^]^ In peripheral tissues, tumors exhibit a greater density of nerve fibers than surrounding normal tissues do, which may suggest that tumors induce the growth of nerve fibers, thereby increasing the number of nerves in the tumor microenvironment (Figure 6b).^[^ ^40^ ^]^ Neurodevelopmental and regenerative processes are aberrantly activated during tumor initiation and progression, leading to a significant increase in intratumoral nerve density and subsequently promoting tumor growth.^[^ ^87^ ^]^ Tumor‐associated neurogenesis has been observed in different cancer subtypes, including prostate cancer, breast cancer, head and neck cancer, and pancreatic cancer. Peripheral tumors attract neural stem cells from the subventricular zone to prostate and breast tumors. These neural stem cells cross the blood–brain barrier and migrate in the bloodstream, subsequently infiltrating the tumor to promote intratumoral neurogenesis and differentiation into adrenergic neurons. Newly formed adrenergic neurons further support and promote cancer progression and metastasis.^[^ ^42^ ^]^ In p53‐deficient head and neck cancer cells, microRNAs shuttle through extracellular vesicles to sensory neurons, inducing the differentiation of adrenergic neurons and thereby promoting tumor progression. Granulocyte colony‐stimulating factor (G‐CSF) is a neurotrophic factor found to support the survival of autonomic nerves in a prostate cancer mouse model.^[^ ^88^ ^]^ In pancreatic cancer, the upregulation of neurotrophic factors such as NGF and BDNF is associated with sympathetic nerve innervation and the enrichment of norepinephrine in the local tumor microenvironment.^[^ ^89^ ^]^ Cancer cells secrete neurotrophic growth factors such as neurotrophin (NT) and axon guidance molecules, nourishing the surrounding nerves locally and promoting neurite outgrowth, which are crucial driving factors for axonalogenesis within tumors.

### 4.3 Neurological Dysfunction Caused by Tumor Treatment

Additionally, conventional standard therapies for treating tumors in clinical practice, especially radiation therapy and chemotherapy, may adversely affect the nervous system. Not only do they directly lead to tissue damage in the central nervous system, such as white matter injury and hippocampal shrinkage, but they also trigger cognitive impairments (Figure 6c). These cognitive impairments manifest as deficits in memory, attention, and multitasking abilities, which are associated with disrupted neural communication and network connectivity. This disruption arises from dysfunctions in neural stem and progenitor cell populations induced by radiotherapy and chemotherapy,^[^ ^90^ ^]^ imbalances in hippocampal neuroregulation,^[^ ^91^ ^]^ disturbances in myelin phospholipid homeostasis and plasticity,^[^ ^92^ ^]^ and synaptic disruption. This prompted us to contemplate how to regenerate neural stem cells and progenitor cell populations, showing promise in early clinical studies addressing cancer treatment‐related cognitive impairments. Additionally, cancer treatments can lead to peripheral neuropathy, characterized by muscle weakness, sensory loss, or pain, as well as dysfunctions in the autonomic nervous system.^[^ ^93^ ^]^ In particular, chemotherapy drugs may affect the autonomic nervous system, resulting in gastrointestinal symptoms such as nausea, vomiting, constipation, and diarrhea.^[^ ^94^ ^]^ A thorough understanding and further investigation of the impact and mechanisms of cancer on neurons can not only halt the malignant cycle of neuro‐cancer interactions but also safeguard the integrity of neural function and impede tumor progression while also aiding in the discovery of potential therapeutic targets.

## 5 Treatment Strategies Based on the Interaction between Nerves and Tumors

Understanding the close connection between the nervous system and cancer progression inspires us to explore potential therapeutic avenues for cancers such as brain cancer, prostate cancer, and pancreatic cancer from new perspectives.^[^ ^95^ ^]^ Targeting the interaction between neurons and cancer cells may become a future clinical treatment strategy. Here, we introduce some ongoing or promising treatment strategies (**Table** **1**).

### 5.1 Targeting Neuroregulatory Substances to Inhibit Tumor Progression

Neural innervation plays a significant role in the progression of various cancers, and targeting neurosecretory factors to block neural input is an evident therapeutic pathway. As previously mentioned, nerves accelerate tumor progression through neurotransmitters such as glutamate, norepinephrine, and acetylcholine. By targeting neurotransmitter regulation in the nervous system, some commonly used clinical neuroregulatory drugs, such as dopamine, β‐adrenergic, and glutamate receptor modulators, can be considered.^[^ ^96^ ^]^ Studies have shown an association between the use of β‐blockers and improved prognosis in cancer patients.^[^ ^97^ ^]^ However, it is critical to note that therapeutic strategies for CNS tumors must account for the limitations of the blood–brain barrier on drug penetration, whereas neural targeting in PNS tumors may rely more on local microenvironmental interactions or systemic autonomic regulation. For example, the efficacy of β‐blockers in gliomas may be constrained by their limited CNS penetration, whereas in breast cancer, these agents could exert systemic antitumor effects by suppressing sympathetic nerve activity. This highlights the need for tailored pharmacologic approaches that consider anatomical and physiological barriers unique to different tumor types. However, when considering the use of neuroregulatory drugs for cancer treatment, it is important to note that these drugs lack tumor specificity and may have potential toxicity at effective doses. The therapeutic window may also be limited by the correlation between normal neural and organismal signaling pathways, necessitating the development of more targeted treatment methods for the interaction between neurons and cancer.

**Figure 8:**
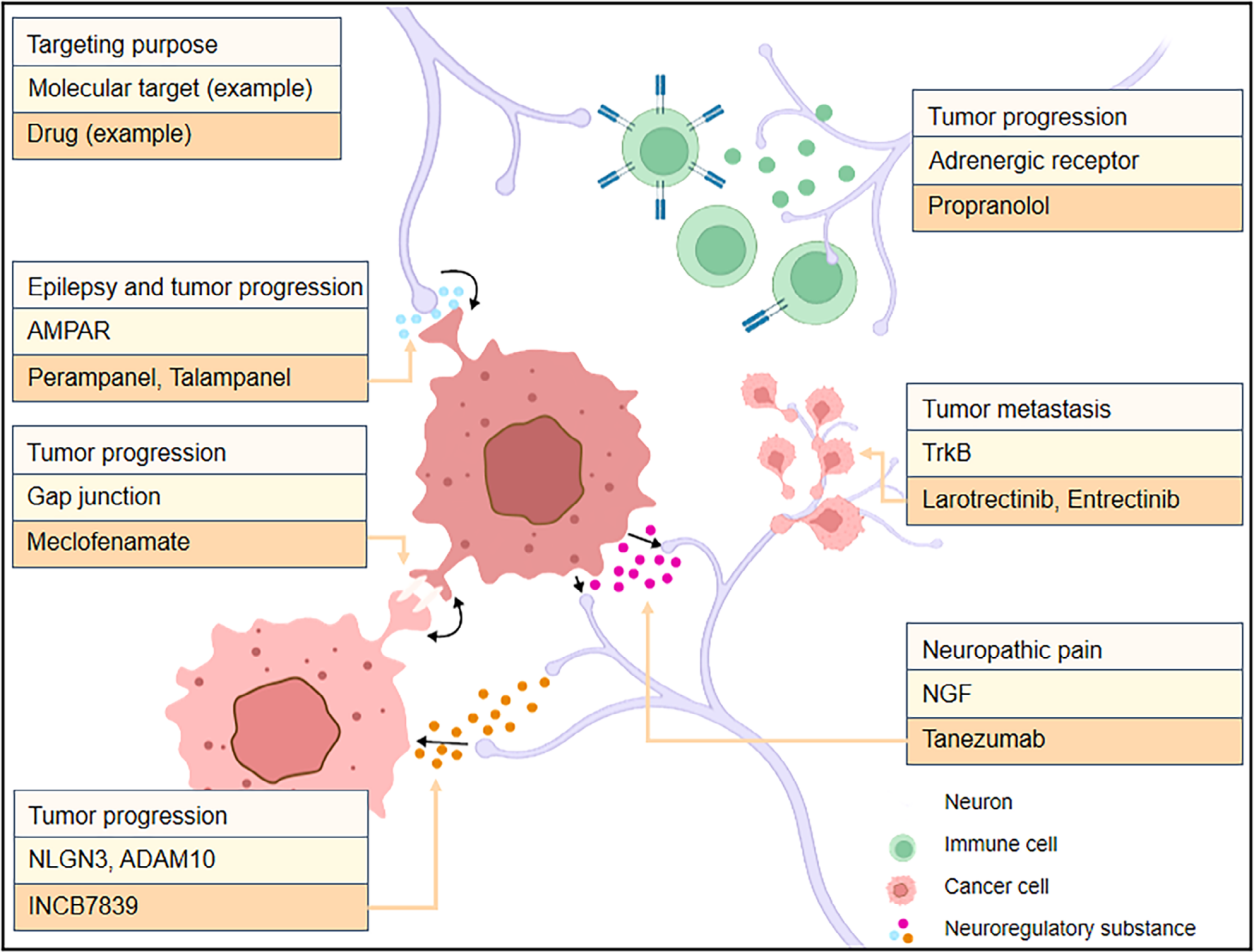
Therapeutic targets and example drugs that target neural regulation of tumor progression and symptoms. This schematic summarizes key molecular targets and corresponding drugs designed to disrupt neural–tumor interactions and associated pathological processes. Annotations are provided below for each molecular target, and the corresponding therapeutic drugs are shown in the figure. AMPAR: A glutamate receptor that mediates excitatory synaptic input to glioma cells. AMPAR antagonists Perampanel and Talampanel inhibit glioma‐associated epilepsy and tumor progression by blocking glutamatergic signaling; Gap junctions: Intercellular channels facilitating communication among tumor cells. Meclofenamate is a gap junction inhibitor that suppresses tumor microtube formation and progression. NLGN3 is a synaptic adhesion molecule shed by neurons that promotes glioma growth. The metalloprotease ADAM10 cleaves NLGN3; INCB7839 is an ADAM10 inhibitor currently in clinical trials to block this pathway. Adrenergic receptors are receptors for catecholamines released by sympathetic nerves, which promote tumor progression. Propranolol, a β‐adrenergic receptor blocker, has demonstrated efficacy in reducing tumor growth; TrkB: A receptor for BDNF involved in tumor metastasis. Larotrectinib and entrectinib are Trk inhibitors that induce cancer cell death and inhibit metastatic spread. NGF is a neurotrophin that drives neuropathic pain associated with perineural invasion in cancers. Tanezumab is a monoclonal antibody against NGF that is used to alleviate cancer‐related neuropathic pain.

For peripheral secretory factors such as BDNF, NGF, and NLGN3, targeting NGF with the NGF monoclonal antibody tanezumab to silence NGF signaling can reduce cancer pain and potentially have antitumor effects. Currently, an ADAM10 inhibitor (INCB7839) is being tested in phase I trials for pediatric high‐grade gliomas to inhibit soluble NLGN3 cleavage, thereby inhibiting glioma cell proliferation and progression, with plans for trials in adult glioblastoma patients (**Figure** **8**). Another clinical trial involved the use of meclofenamate (a gap junction inhibitor) in combination with temozolomide chemotherapy to inhibit tumor microtube‐dependent intercellular communication in recurrent adult glioblastomas (Figure 8). Compared with neurotransmitters, there are fewer drugs that regulate nearby secretion signals from neurons. However, leveraging the efficacy and safety of existing drugs and innovatively applying them to cancer treatment can bring new vitality to traditional cancer therapy. Additionally, brain tumors are less responsive to conventional chemotherapy because of the presence of the blood–brain barrier, while many neuroregulatory drugs can easily penetrate the blood–brain barrier, making this treatment strategy particularly attractive for brain tumors.

Furthermore, early surgical denervation has shown promising therapeutic effects in experimental gastric and prostate cancer mouse models, effectively inhibiting tumor progression. Moreover, denervation strongly inhibited the growth of basal cell carcinoma in mouse models.^[^ ^98^ ^]^ However, the full consequences of complete nerve removal are not yet fully understood and are difficult to control, making its complete application in clinical practice challenging. Nevertheless, when there is a high risk of tumor resection, local denervation through minimally invasive surgery may have the potential to inhibit cancer progression.

### 5.2 Disrupting Electrical Hyperexcitability to Achieve Dual Inhibition of Epilepsy and Tumor Progression

The malignant behavior of aggressive gliomas, such as glioblastoma (GBM), and brain metastases is closely linked to dynamic electrophysiological interactions with neurons. Tumor cells integrate into neural circuits through neurotransmitter receptors (e.g., AMPA and NMDA) and ion channels expressed on their surface, hijacking neuronal activity to fuel growth. For example, glioma cells express calcium‐permeable AMPA receptors (GluA2‐lacking subtypes), which trigger calcium influx upon presynaptic glutamate release. This calcium signaling pathway activates downstream oncogenic pathways (e.g., the PI3K‐mTOR and NF‐κB pathways), promoting tumor proliferation and further upregulating AMPAR expression—a self‐reinforcing feedforward loop (Figure 8).^[^ ^22^ ^]^ Additionally, gliomas remodel peritumoral neural circuits by secreting glutamate and microenvironmental proteins such as TSP‐1. Excess glutamate downregulates the KCC2 cotransporter in neurons, reducing inhibitory GABAergic tone, whereas TSP‐1 drives aberrant synaptogenesis and dendritic sprouting. These changes create hyperexcitable neural networks, exacerbating tumor‐associated epileptiform activity and forming a vicious cycle: neuronal hyperexcitability drives tumor growth, whereas tumor‐derived factors destabilize neural circuit stability.

Noncompetitive AMPAR antagonists, such as perampanel, have dual potential in suppressing both epilepsy and tumor growth in preclinical models. For example, in patient‐derived xenograft mouse models, perampanel significantly reduces glioma cell proliferation and invasion by inhibiting AMPA receptor activity at neuron–glioma synapses. Additionally, perampanel suppresses the formation of TMs, thereby disrupting functional intercellular tumor networks. In clinical research, a phase II trial (PerSurge trial, EU‐CT number: 2023–503938–52–00) evaluated the efficacy of perampanel in recurrent glioblastoma. The coprimary endpoints included a tumor connectivity score (based on single‐cell transcriptomics) and AI‐supported MRI analyses of tumor growth dynamics.^[^ ^99^ ^]^ Preliminary data indicate that perampanel reduces seizure frequency and delays tumor progression by inhibiting neuron–tumor synaptic connectivity. Notably, as an EMA/FDA‐approved antiepileptic drug, perampanel has shown good tolerability and efficacy in the treatment of brain tumor‐related epilepsy. Observational studies have reported that low‐dose perampanel (2–4 mg per day) reduces seizure frequency in more than 75% of patients, with 50% achieving seizure freedom.^[^ ^100^ ^]^

Furthermore, perampanel's ability to penetrate the blood–brain barrier and long half‐life (≈105 h) make it an ideal candidate for glioma therapy. In mice with neurofibromatosis type 1 (NF1), neurons with NF1 gene mutations exhibit excessive excitation. Lamotrigine, an FDA‐approved antiepileptic drug, can inhibit this overexcitation, thereby halting tumor growth in mice.^[^ ^101^ ^]^ The therapeutic potential of additional antiepileptic drugs in tumor treatment awaits further research and clinical trials.

Despite these advancements, critical challenges persist. Tumor plasticity may drive adaptive activation of alternative ion channels (e.g., TRPV1) under sustained AMPAR inhibition, but exploration of combination therapies is needed. Improving blood–brain barrier penetration through nanocarrier technologies (e.g., lipid‐encapsulated drugs) is essential for enhancing drug delivery to the central nervous system. Additionally, standardizing electroencephalography (ECoG) biomarkers, such as high‐frequency oscillations, across multicenter trials is crucial to validate their clinical utility.

### 5.3 Targeting Sensory Nerves for the Treatment of Neuropathic Pain

In turn, tumor invasion around nerves can reshape neural structures, inducing cancer‐related pain, partly through the action of neurotrophic factors and neuroregulatory proteins. For example, NGF activation of sensory afferent nerves from the dorsal root ganglion is most directly associated with pancreatic cancer pain.^[^ ^53^ ^]^ Therefore, targeting these signaling pathways, such as the vascular endothelial growth factor (VEGF) and chemokine signaling pathways involved in PNI and cancer pain, can not only control tumor progression but also effectively suppress pain.^[^ ^102^ ^]^ According to cancer neuroscience research, treatments targeting neuropathic pain can not only alleviate symptoms and relieve the suffering of cancer patients but also potentially promote control over cancer progression.

Considering that the signaling proteins secreted by upstream pain regulators can promote tumor growth, these neuronal signaling pathways may be effective therapeutic targets. Some patients with advanced cancer experience severe and refractory pain, which may be attributed to these potential neurotrophic mechanisms. Therefore, therapeutic strategies targeting these tumor nerve signals have the potential to control neuropathic cancer pain and fight cancer. As mentioned earlier, the anti‐NGF monoclonal antibody tanezumab can be used to treat painful bone metastasis (Figure 8). In addition, targeting the TRPV1 receptor downstream of NGF can promote the sensitization of sensory neurons, which is also a potential strategy.

Notably, while cancer itself can cause neuropathic pain, treatments such as chemotherapy‐induced peripheral neuropathy can also induce pain. Dorsal root ganglia, which lack a blood–brain barrier, are susceptible to neurotoxic injury, which is believed to contribute to many sensory symptoms associated with chemotherapy‐induced peripheral neuropathy.^[^ ^103^ ^]^ Additionally, sensory neurons in the dorsal root ganglia play a role in the development and progression of certain cancers. In mouse models, even at the PanIN‐2 stage, the nervous system undergoes neural infiltration, inflammation, and neuronal damage. Using neonatal capsaicin injection to ablate sensory neurons can prevent neural infiltration and neuronal damage, indicating that sensory neurons transmit inflammatory signals induced by oncogenic Kras to the central nervous system.

### 5.4 Targeting PNI for the Inhibition of Tumor Metastasis along Nerves

The migration of primary tumors via blood vessels and lymphatic channels to form secondary tumors poses a significant challenge in cancer therapy. Nerves significantly contribute to this process by acting not only as physical conduits^[^ ^104^ ^]^ but also as mediators of dissemination via blood vessels or lymphatics.^[^ ^105^ ^]^ Studies have demonstrated an association between the PNI and metastasis.^[^ ^106^ ^]^ Neurotrophic factors such as NGF and BDNF are increasingly recognized as controllers of the irregular creation of blood vessels.^[^ ^107^ ^]^ NGF has been shown to inhibit tumor growth, metastasis, and angiogenesis in xenografted mice with breast cancer.^[^ ^108^ ^]^

Metastasis culminates in the successful colonization of distant organs, with the neurotrophic receptor TrkB accelerating metastatic spread. The inhibition of TrkB (e.g., larotrectinib or entrectinib) can induce cancer cell death in locally advanced malignant tumors before distant organ colonization. As mentioned earlier, the nervous system plays a role in breast cancer brain metastasis, where the neurotransmitter glutamate activates NMDAR signaling in breast cancer cells, promoting successful brain colonization. Mice with reduced NMDAR GluN2B subunits exhibit decreased growth of brain metastases and a longer period without brain metastases.^[^ ^25^ ^]^ In cases of metastatic dissemination, treatment strategies targeting the mechanisms of metastatic growth, such as NMDAR receptor antagonists such as memantine, may be viable options for patients with breast cancer brain metastasis.

The nervous system also plays a significant role in regulating breast cancer metastasis. In systemic injection and in situ breast cancer mouse models, chronic stress induction significantly increases tumor colonization and metastasis to distant tissues.^[^ ^109^ ^]^ Blocking β‐adrenergic receptors in in situ breast cancer models can eliminate the stress‐induced prometastatic transition. Preclinical studies have shown that the use of propranolol, a β‐blocker in early breast cancer, can reduce interstitial polarization in tumors, promote the infiltration of immune cells, and reduce biomarkers related to metastasis potential.^[^ ^110^ ^]^ Stress‐induced neuroendocrine activation significantly increases the metastasis of primary tumors to distant tissues via β‐adrenergic signal transduction, which increases the infiltration of macrophages into the primary tumor parenchyma, thus increasing the expression of metastasis‐promoting genes.^[^ ^109^ ^]^ In conclusion, these potential clinical translations prompt the consideration of therapeutic interventions targeting tumor–neural interactions utilizing neuroactive agents as strategies to inhibit or slow metastatic spread.

## 6 Innovative Technologies Drive a Paradigm Shift in Cancer Neuroscience

Recent advances in cancer neuroscience have delineated neural–tumor crosstalk as a potential therapeutic axis. However, progress toward clinical translation remains limited, largely owing to incomplete circuit mapping, inadequate spatiotemporal resolution, and extensive intertumoral heterogeneity. To address these barriers at both the mechanistic and technological levels, we present three complementary and synergistic approaches: 1) AI‐driven neural circuit decoding, which integrates single‐cell multiomics and in vivo electrophysiology to resolve activity‐dependent protumoral signaling; 2) optogenetic‐based tumor interface engineering, which enables subtype‐specific modulation; and 3) pancancer neural addiction modeling, which combines 3D organotypic cocultures and deep learning to identify conserved neurodevelopmental dependencies. This framework establishes a mechanistic bridge between neural pathway topology and combinatorial targeting strategies, overcoming current limitations in specificity and predictive power.

### 6.1 AI‐Driven Neural Circuit Decoding Revolution

Artificial intelligence (AI) is revolutionizing the way we decode neural circuitry, providing unprecedented capabilities to analyze and interpret the complex dynamics of tumor‐associated neural networks. Traditional methods such as electrophysiology and histology, while invaluable, are limited in spatial resolution, throughput, and contextual integration. In contrast, AI‐driven approaches can integrate large‐scale datasets, including high‐resolution imaging, spatial transcriptomics, single‐cell omics, and functional recordings, to construct multiscale, data‐rich representations of tumor–nerve interactions. This multiscale integration allows AI to bridge spatial and temporal scales—from macroscopic tumor innervation patterns to microscopic synaptic connectivity and intracellular signaling—offering a systems‐level understanding of tumor–nerve crosstalk.

Recent efforts have demonstrated that deep learning algorithms, particularly graph neural networks, convolutional neural networks, and attention‐based models, can identify subtle neural activation patterns, predict neural subtype classification, and infer functional synaptic architectures from noisy or incomplete data sources. For example, the combination of spatial transcriptomic maps and calcium imaging datasets allows for AI‐enabled reconstruction of tumor‐innervating circuits, revealing how distinct neuronal populations influence tumor behavior through activity‐dependent mechanisms.^[^ ^111^ ^]^ Moreover, integrative frameworks that couple unsupervised dimensionality reduction techniques (e.g., UMAP, t‐SNE) with supervised learning models have shown promise in identifying latent features associated with tumor‐promoting neural inputs.^[^ ^111^ ^]^ These models increasingly incorporate interpretable architectures, such as attention mechanisms and SHAP‐based feature attribution, to identify the most influential neural inputs or tumor regions, thereby enhancing biological plausibility and hypothesis generation. Such methods have already been applied in preclinical models of glioma and pancreatic cancer to delineate peritumoral neural remodeling, and they hold promise for cross‐cancer applications where conserved neural programs may drive tumor progression.

Building on these capabilities, the next frontier lies in dynamically interfacing AI models with experimental neuromodulation tools. Closed‐loop systems that integrate AI‐based neural decoding with optogenetic stimulation or neuromodulation platforms may enable real‐time interrogation of tumor‐associated circuits. These systems can dynamically predict tumor responses to neural activity or therapy‐induced remodeling, ultimately informing the design of personalized, neurotargeted cancer interventions. In summary, AI‐driven neural decoding is shifting the paradigm of cancer neuroscience from static structural mapping to dynamic, predictive, and intervention‐oriented systems. Its integration with neuroscience, oncology, and bioengineering will be pivotal in enabling precision, circuit‐level modulation of tumor progression.

### 6.2 Optogenetic Tumor Regulation Technology

Building on insights gained through AI‐based neural decoding, optogenetic technologies have emerged as powerful tools for directly manipulating neural activity within tumor‐associated circuits. Recent advances in cancer neuroscience have shown that cancer cells use neural circuits and neuronal electrical activity to promote their growth, survival, and spread. Optogenetics has become a powerful approach for controlling neuronal activity with high temporal and spatial precision, enabling detailed studies of nerve–tumor interactions.

For example, in gastric cancer, sensory nerves dependent on NGF expand and form direct connections with tumor cells. Using optogenetics to activate these nerves increases calcium signaling in cancer cells, which drives tumor growth and metastasis. Blocking these nerves or their signals can suppress tumor progression.^[^ ^45^ ^]^ In brain tumors such as H3K27M‐mutant diffuse midline gliomas, optogenetics combined with electrophysiology has revealed that GABAergic neurons form synapses with tumor cells, where the neurotransmitter GABA induces depolarization and proliferation in a subtype‐specific manner.^[^ ^112^ ^]^ In small‐cell lung cancer, tumor cells themselves exhibit electrical excitability, and optogenetic tools have elucidated how such activity increases tumor aggressiveness and metabolic demands.^[^ ^113^ ^]^ Furthermore, advanced light‐controlled systems now allow the manipulation of intracellular protein signaling, offering refined insight into the tumor–nerve communication axis.^[^ ^114^ ^]^

In parallel with these mechanistic insights, optogenetic engineering platforms have evolved toward translational relevance. Emerging technologies such as wireless transcranial systems, biodegradable optoelectronic implants, and integrated microLED–ECoG arrays now enable minimally invasive, spatially precise, and chronically stable neural modulation.^[^ ^115^ ^]^ These innovations lay a robust technical foundation for mapping and manipulating tumor‐associated neural circuits in situ, with promising implications for future therapeutic strategies.

Overall, optogenetics not only facilitates the modulation of tumor‐associated neural activity but also enables the exploration of the electrophysiological properties of tumor cells, thereby offering deeper insights into the complex tumor–nerve interplay. Nevertheless, its direct application in tumor neurobiology remains limited, with most current studies focusing primarily on fundamental neural circuitry or preliminary investigations into neuroimmune regulatory mechanisms. Given the intricate neural remodeling and cellular heterogeneity within the tumor microenvironment, integrating optogenetic technologies into platforms for real‐time visualization, precise neuromodulation, and even personalized intervention holds great promise. This review thus proposes that fostering interdisciplinary research at the interface of optogenetics and tumor‐associated neural regulation may become a pivotal direction for future exploration in this emerging field.

### 6.3 Pancancer Patterns of Neural Addiction Revealed by Computational Modeling

While neurotrophic signaling is increasingly recognized in tumor progression, the systematic characterization of neural addiction heterogeneity across malignancies remains elusive. Despite recent efforts in pancancer genomic mapping, such as those from the PCAWG consortium, most analyses have focused on canonical driver mutations, with limited attention given to tumor–nerve crosstalk or neural dependency traits.^[^ ^116^ ^]^ Emerging pancancer analyses employing machine learning have begun to deconvolute conserved and cancer‐type–specific neural addiction signatures through multimodal integration of neurotransmitter receptor landscapes, axon guidance molecule dynamics, and tumor‐nerve spatial colocalization patterns.^[^ ^117^ ^]^ To further advance this research, we propose a computational framework for the development of a predictive model of neural addiction signatures across cancer types.

To characterize the diversity of neural dependency across cancer types and develop a predictive model of neural addiction traits, it is essential to integrate multidimensional data with artificial intelligence technologies. The system first aggregates multiomics datasets, including transcriptomics, epigenomics, proteomics, and spatial transcriptomics, with a focus on the expression profiles of neurosignaling molecules (e.g., neurotransmitter receptors, axon guidance cues, and synaptic proteins).^[^ ^118^ ^]^ Single‐cell or single‐nucleus sequencing is employed to dissect the heterogeneity of neuron–cancer cell interactions within the tumor microenvironment, while clinical data (e.g., treatment responses, survival outcomes, and neuro‐comorbidities) are correlated to define neuro‐dependent subtypes.^[^ ^119^ ^]^ Key features associated with neurodevelopmental processes (e.g., synaptogenesis, axonal growth) or activity‐dependent pathways (e.g., calcium signaling‐mTOR, BDNF/TrkB) are identified via dimensionality reduction techniques (UMAP, PCA) and graph neural networks to construct a spatially resolved neuro–tumor interaction network.^[^ ^120^ ^]^

An ensemble learning framework (e.g., XGBoost, deep survival networks) is subsequently used to train a predictive model, quantifying the neural addition score (NAS) for different cancer types. Transfer learning is employed to enhance cross‐cancer generalization, and interpretability algorithms (e.g., SHAP, attention mechanisms) are used to uncover core drivers such as NLGN3 and GRIN2B.^[^ ^27^, ^28^ ^]^ Model validation is supported by preclinical experiments, such as evaluating tumor proliferation under neuron‐activity dependency in optogenetic models or using in vivo calcium imaging to verify synaptic connection strength. In the clinical translation phase, patients are stratified on the basis of the NAS to guide precision therapies targeting neural checkpoints (e.g., the AMPA receptor inhibitor perampanel).

Finally, a dynamic learning system is introduced by incorporating federated learning to continuously integrate longitudinal electrophysiological data (e.g., ECoG recordings) and therapy‐induced neural remodeling, thereby enhancing model adaptability. A visual AI platform is developed to embed neural addiction features into clinical decision‐making, providing navigational support for personalized neuromodulatory strategies. This integrative framework, which bridges computational oncology and neuroscience, aims to uncover the neurodependence of tumors and advance precision therapeutics that target neural circuits.

Recent advances in artificial intelligence, optogenetics, and computational modeling have introduced powerful tools for dissecting tumor–neural interactions, enabling the identification of key neural components, the characterization of cancer type‐specific neural dependencies, and the precise modulation of neuronal activity. These innovations not only provide mechanistic insights into how neural circuits influence tumor progression but also pave the way for the development of targeted neuromodulatory strategies across diverse malignancies.

## 7 Conclusion and Outlook

The emergence of cancer neuroscience has fundamentally reshaped our understanding of tumor biology, establishing the nervous system as a critical regulator of malignancy. Key discoveries—such as activity‐dependent neurotransmitter release and synapse‐like contacts between neurons and cancer cells—have revealed that neural inputs not only contribute to tumor progression but also represent targetable vulnerabilities. Although initial attempts to therapeutically disrupt neural–tumor crosstalk (e.g., NGF/TrkA or semaphorin pathway blockade) have shown limited efficacy when used alone, their combination with conventional therapies (chemotherapy, radiotherapy, or immunotherapy) offers a promising strategy through synergistic mechanisms, a concept now referred to as neural checkpoint modulation.

However, translating these insights into clinical benefits requires overcoming three major challenges. First, mechanistic specificity remains difficult because of the pleiotropic functions of key neurodevelopmental molecules such as Netrin‐1, necessitating context‐sensitive targeting strategies to avoid off‐tumor effects. Second, neural feedback loops that promote tumor plasticity call for adaptive treatment strategies. Tools such as in vivo neural activity mapping and optogenetic modulation may support real‐time therapeutic adjustments. Third, safe and effective delivery remains a bottleneck; spatiotemporally controlled systems—such as nanoformulations of neurotransmitter modulators or optogenetic actuators—could increase on‐target efficacy while minimizing disruption to normal neural function.

In the future, interdisciplinary convergence will be essential to address these barriers. AI‐based neural circuit modeling, which integrates spatial transcriptomics, functional imaging, and machine learning,^[^ ^121^ ^]^ is beginning to reveal the organization and dynamics of tumor‐associated neural networks. Combined with single‐cell neurotranscriptomics and real‐time calcium imaging, these tools could define malignancy‐specific neural activation patterns, or “neural addiction” signatures, to guide personalized neuromodulatory therapies. Redefining response criteria to include neuromodulation biomarkers and incorporating functional neuroimaging into clinical trial design will be critical for translational progress.

As the field evolves, cancer neuroscience is poised to complement existing pillars of oncology—antiangiogenic, cytotoxic, and immunotherapeutic strategies—by introducing neural modulation as a new treatment axis. The long‐term vision includes the development of closed‐loop neuromodulatory systems capable of detecting and intercepting tumor‐associated neural activity in real time. By decoding the electrical signals of tumor–nerve interactions and targeting the circuits that support malignancy, this emerging discipline holds the potential to transform neural regulation into a powerful tool for precision oncology.

## Conflict of Interest

The authors declare no conflict of interest.
